# Noradrenaline-trajectory phenotypes in septic shock: derivation and external validation in two independent cohorts

**DOI:** 10.1186/s40635-026-00910-8

**Published:** 2026-06-22

**Authors:** Gargi Yonatan, Amir Cohen, Dorit Stein, Ori Levi, Dor Cohen, Julia Klein, Hamutal S. Taube, Jacob Vine, Maxim Glebov, Teddy Lazebnik, Mor Saban, Yael Haviv, Eran Segal

**Affiliations:** 1 Departments of Anesthesiology and Intensive Care, Sheba Medical Center, Ramat Gan, Israel; 2 Department of Intensive Care, Sheba Medical Center, Ramat Gan, Israel; 3 Departments of Nutrition and Intensive Care, Sheba Medical Center, Ramat Gan, Israel; 4 Intensive Care Unit, Sheba Medical Center, Ramat Gan, Israel; 5 Universidad Favaloro, Buenos Aires, Argentina; 6 Department of Information Systems, University of Haifa, Haifa, Israel; 7 Department of Computing, Jonkoping University, Jonkoping, Sweden; 8 Gertner Institute for Epidemiology and Healthcare Research, Gray Faculty of Medical and Health Sciences, Tel Aviv University, Tel Aviv-Yafo, Israel; 9 Department of Intensive Care, Maaynei Hayeshua, Bnei Brak, Israel

**Keywords:** Septic shock, Phenotypes, Noradrenaline trajectories, Dynamic time warping

## Abstract

**Background:**

Septic shock is heterogeneous, and noradrenaline (NA) requirements evolve over time in ways that reflect vascular responsiveness and shock biology. Whether long-horizon NA dose-trajectory phenotypes are reproducible across healthcare systems and identifiable early in the clinical course remains uncertain.

**Methods:**

We retrospectively analyzed 1111 adults with septic shock at Sheba Medical Center (Israel) and 9343 adults from MIMIC-IV. Hourly NA infusion trajectories were reconstructed for 10 days. A fully prespecified clustering pipeline combining static-feature K-means with dynamic time warping refinement was derived in Sheba and applied unchanged to MIMIC-IV. The primary outcome was 90-day mortality; 30-day mortality was analyzed as a supportive secondary outcome. Secondary analyses included feature interpretability, multivariable landmark Cox models (24–144 h), and early phenotype prediction using exposure features available at 24–96 h.

**Results:**

Five stable phenotypes emerged in Sheba and six in MIMIC-IV. Despite this numerical difference and somewhat more prolonged high-dose exposure in Sheba sustained/late-escalating patterns, both cohorts exhibited the same core families of trajectory shapes with preserved mortality gradients (90-day mortality 37–89% in Sheba; 16–69% in MIMIC-IV). Mortality gradients were preserved at both 30 and 90 days across cohorts. Across cohorts, exposure-persistence features—particularly time to maximal NA dose and cumulative NA burden during days 2–4—were key determinants of phenotype structure and mortality. Early-prediction models identified final phenotypes using 24-h data with 85% accuracy in Sheba and 86% in MIMIC-IV, improving by 48 h to 89% and 88%, respectively; high-confidence assignments achieved approximately 91%–95% accuracy.

**Conclusions:**

Long-horizon NA dose-trajectory phenotypes are clinically interpretable hemodynamic exposure/response patterns, prognostically coherent, and externally reproducible. Their defining features were detectable within the first 24 h and showed stronger discrimination by 48 h. At present, these phenotypes should be interpreted as descriptive hemodynamic-response patterns that may support dynamic risk stratification, prognostic enrichment for future trials, and hypothesis generation; whether early phenotype identification can improve management or outcomes requires prospective evaluation.

**Supplementary Information:**

The online version contains supplementary material available at 10.1186/s40635-026-00910-8.

## Introduction

Septic shock is a life-threatening manifestation of sepsis, characterized by profound hypotension and organ hypoperfusion, with mortality rates often exceeding 40% [1]. NA is widely recommended as the first-line vasopressor to restore adequate mean arterial pressure in septic shock [2], and its dosing requirements are often interpreted as a marker of shock severity [3–5]. Patients who require high-dose NA have particularly poor outcomes [6, 7].

However, clinical responses to NA vary widely: some patients rapidly stabilize and can be weaned off vasopressors within 24–48 h, whereas others require escalating or sustained support, or develop refractory vasoplegia despite increasing doses [8–10], and often progress to multiorgan failure. These divergent patterns underscore the biological and clinical heterogeneity of septic shock and motivate the use of phenotyping approaches grounded in dynamic vasopressor behavior rather than isolated measurements.

Prior observational studies have shown that prolonged or escalating NA exposure is strongly associated with increased mortality and organ failure [11, 12], and that an inability to taper vasopressors in the first several days is a powerful predictor of poor prognosis [12].

Unsupervised machine learning methods have proven to be powerful methods for uncovering phenotypes derived from temporal clinical data [8, 9]. Earlier approaches primarily clustered patients using static features, yielding phenotypes defined mainly by organ-failure patterns [13–17].

More recent approaches have incorporated time-series information to better capture the dynamic evolution of critical illness [18, 19]. For example, Shen et al. showed that the mortality impact of vasoactive drug dose and duration varies markedly across sepsis phenotypes [20], underscoring the clinical relevance of vasopressor time-course patterns. However, these studies continue to treat vasopressor use as an exposure rather than as a defining dynamic phenotype. This motivates the need to characterize vasopressor trajectories themselves as reproducible shock phenotypes.

More recent work has focused on vasopressor-dose trajectories. For example, Yang et al. (2025) applied group-based trajectory modeling to NA dosing over the first 4 days of septic shock and identified three distinct dose-trajectory phenotypes: low, intermediate, and high [12]. The high-dose group had worse baseline severity and mortality.

These studies show that clustering in the time domain, not just the static domain, can reveal meaningful septic-shock phenotypes with potential relevance for dynamic risk stratification and later supportive decision-making.

Classifying septic-shock patients according to their NA dose trajectories may improve dynamic risk stratification after the initial resuscitation phase, may help characterize persistence versus resolution of hemodynamic support needs, and may reduce physiologic heterogeneity in observational studies and interventional trials. However, it remains unknown whether long-horizon NA-trajectory phenotypes are reproducible across healthcare systems and whether phenotype-related trajectory signal can already be identified within the first 24 h of shock management, with more robust discrimination by 48 h.

To address these gaps, we pursued two objectives: (1) derive robust NA-trajectory phenotypes in a large single-center cohort using a transparent and reproducible clustering pipeline, and (2) perform external validation of the identical, fully prespecified pipeline in the MIMIC-IV database, representing a different continent, healthcare system and ICU practice environment.

## Methods

### Study design and cohort

The derivation cohort comprised 1111 adult ICU admissions with septic shock at Sheba Medical Center (Israel) between 2012 and mid-2025, defined by a clinical septic shock diagnosis with lactate > 2 mmol/L prior to or at ICU admission and active NA infusion. NA infusion trajectories were reconstructed and standardized to norepinephrine base-equivalent µg/kg/min using first-day recorded weight. In the Sheba cohort, norepinephrine was administered as Levophed or equivalent formulations, which are supplied as bitartrate salts but labeled in base-equivalent terms; thus, doses were analyzed on a harmonized base-equivalent scale [21].

The external validation cohort was drawn from MIMIC-IV v2.2 (2008–2019) at Beth Israel Deaconess Medical Center (Boston, USA) [22]. Septic shock was identified using the published Sepsis-3 computable phenotype (mimiciv_derived.septic_shock); to enable trajectory construction, we additionally required documented NA infusion records. For cross-cohort comparability, MIMIC-IV norepinephrine doses were likewise interpreted on a base-equivalent µg/kg/min scale. The final validation cohort included 9343 ICU stays (Supplement Fig. S1).

### Exposure construction

Medication administration records were converted into hourly NA infusion time-series over a 10-day horizon.

### Feature engineering

From each hourly NA series, we derived features capturing exposure magnitude and persistence (AUC-based measures), escalation timing and intensity, time-above clinically relevant dose thresholds, de-escalation kinetics, and post-peak rebound behavior. Full feature definitions and rationale regarding high-dose cutoffs are provided in the Supplement.

### Clustering workflow

We applied a prespecified two-stage clustering framework combining feature-based K-means with DTW-based refinement of NA time-series trajectories. In this framework, the primary coarse cluster structure was learned in the engineered feature space, whereas DTW refinement was used to improve alignment with similarity in the underlying temporal trajectories. We did not apply unsupervised feature reduction or formal de-correlation before clustering. This was intentional: the feature library included partially overlapping cumulative AUC and time-over-threshold variables, but also non-AUC trajectory descriptors such as time to maximal dose, post-peak rebound count, slopes, slope-angle changes, and recovery metrics. These variables were retained because they encode complementary and clinically interpretable aspects of exposure magnitude, timing, persistence, escalation, and resolution. Accordingly, the resulting phenotypes should be interpreted as clusters derived within a prespecified norepinephrine exposure–response feature representation, rather than as representation-neutral biological endotypes. Clusters with fewer than 30 patients were absorbed into the most similar larger cluster based on DTW medoid similarity. This minimum-size rule was chosen to favor stability and reproducibility of the final phenotype set, although it may reduce sensitivity for rare but potentially meaningful micro-phenotypes. For interpretability purposes only, phenotypes were renumbered by prognosis and assigned descriptive labels based on their NA dose–time patterns; this step did not influence clustering, feature selection, or external validation.

### Outcomes

The primary outcome was 90-day all-cause mortality. Because longer term mortality may be influenced by baseline comorbidity and post-acute events, 30-day all-cause mortality was also examined as a supportive secondary outcome. Survival was evaluated using landmarked Kaplan–Meier analyses, ΔRMST, and Cox proportional hazards models, with the lowest mortality phenotype as the reference. Sensitivity analyses are detailed in the Supplement.

### Feature importance and outcome association (interpretability)

To identify which trajectory features distinguished phenotypes and contributed to prognosis, we performed an individual-feature interpretability analysis assessing both feature discrimination across clusters and univariable associations with 90-day mortality (HR per 1 SD). Methodological details are provided in the Supplement.

### Landmarked multivariable modeling across time points

We fitted separate Cox models for 90-day mortality at prespecified landmarks (24–144 h) after ICU admission, including age, sex, SOFA, APACHE II, and the top trajectory features. At each landmark, the risk set included only patients alive and still under observation at that time; accordingly, estimates should be interpreted as prognosis conditional on survival to the relevant landmark rather than prognosis from ICU admission. Because the cardiovascular component of the conventional SOFA score incorporates vasopressor use, we additionally performed sensitivity Cox analyses at the 96-h landmark replacing SOFA with a modified SOFA score excluding the cardiovascular component. These 96-h sensitivity analyses were performed in both the Sheba and MIMIC-IV cohorts for both 30-day and 90-day mortality, using a base model that included cluster, age, sex, APACHE II, and the modified non-cardiovascular SOFA score, and an extended model that further adjusted for use of non-norepinephrine vasopressors, inotropic support, and renal replacement therapy by the landmark. Continuous covariates were standardized, with light penalization applied to improve stability. Hazard ratios with 95% CIs were reported for all landmarks (methodological details are provided in the Supplement).

### Internal validation

Internal validation was performed using an event-stratified train/test split, with discrimination assessed by Harrell’s C-index. Additional details are provided in the Supplement.

### External validation

The entire feature-engineering and clustering pipeline was frozen after derivation and applied without modification to the MIMIC-IV dataset.

We evaluated whether NA-trajectory phenotypes could be identified early by training horizon-limited multinomial logistic regression models using data available within the first 24–96 h after ICU admission. For each horizon, models used the top eight Sheba-ranked exposure features computable within that window. Continuous variables were standardized using the Sheba training data.

Because the derivation cohort yielded five phenotypes whereas MIMIC-IV produced six, external labels were harmonized prior to validation by merging two low-dose, early-resolving MIMIC-IV phenotypes into a single minimal/low early resolver class; remaining phenotypes were mapped based on DTW-refined trajectory similarity.

Models were trained exclusively in Sheba using class-balanced multinomial logistic regression with L2 regularization and temperature scaling. Performance was evaluated internally on an independent Sheba test set and externally in the harmonized MIMIC-IV cohort without retraining, using accuracy, macro-F1, Brier score, and correctness of high-confidence predictions (P_max ≥ 0.80).

## Results

### Cohort and cluster structure

A total of 1111 admissions were included in the Sheba derivation cohort and 9343 admissions in the MIMIC-IV validation cohort. Patient demographics, illness severity, mortality, organ-support variables, selected early laboratory markers, major comorbidities, and infection source are summarized for selected matched phenotype pairs in Table 1, with full native-cluster characterization of all five Sheba phenotypes and all six MIMIC-IV phenotypes provided in Supplementary Table S1.

**Table 1:** Condensed clinical characteristics of selected matched norepinephrine-trajectory phenotypes in Sheba and MIMIC-IV

Variable	Sheba low, early resolver (*n* = 610)	MIMIC low, early resolver (*n* = 2205)	Sheba intermediate, gradual wean (*n* = 167)	MIMIC intermediate, gradual wean (*n* = 177)	Sheba intermediate, non-resolver (*n* = 112)	MIMIC intermediate, non-resolver (*n* = 114)
Demographics, severity and mortality						
Age, years	63.0 ± 15.3	67.3 ± 15.1	61.1 ± 14.6	62.6 ± 13.8	62.5 ± 13.6	63.7 ± 15.0
Male sex	344 (56.4%)	1287 (58.4%)	100 (59.9%)	106 (59.9%)	72 (64.3%)	69 (60.5%)
APACHE II	29.3 ± 8.8	29.9 ± 8.2	31.5 ± 7.1	34.6 ± 7.4	30.1 ± 6.5	29.1 ± 9.0
SOFA (24 h max)	12.5 ± 3.9	11.1 ± 3.5	14.9 ± 3.6	13.0 ± 2.9	14.1 ± 3.6	11.7 ± 3.8
SOFA without cardiovascular component	9.7 ± 3.5	8.2 ± 3.4	11.3 ± 3.5	10.1 ± 2.8	10.8 ± 3.2	9.4 ± 3.5
90-day mortality (96-h landmark)	37.0%	18.7%	53.5%	55.1%	89.3%	69.3%
30-day mortality (96-h landmark)	23.1%	17.1%	39.6%	50.4%	74.1%	66.7%
Mechanical ventilation (admission)	495 (81.1%)	1731 (78.5%)	146 (87.4%)	156 (88.1%)	107 (95.5%)	88 (77.2%)
Acute kidney injury (admission)	298 (48.9%)	1403 (63.6%)	93 (55.7%)	150 (84.7%)	54 (48.2%)	83 (72.8%)
RRT any ICU	164 (27.4%)	405 (18.4%)	99 (59.3%)	101 (57.1%)	67 (59.8%)	73 (64.0%)
Cardiogenic shock	17 (2.8%)	305 (13.8%)	9 (5.4%)	41 (23.2%)	9 (8.0%)	30 (26.3%)
Any non-NE vasopressor any ICU	434 (72.5%)	1234 (56.0%)	163 (97.6%)	171 (96.6%)	110 (98.2%)	96 (84.2%)
Any inotrope any ICU	140 (23.4%)	495 (22.4%)	77 (46.1%)	99 (55.9%)	49 (43.8%)	49 (43.0%)
Peak lactate first 24 h, mmol/L	3.0 [2.0, 5.16]	3.4 [2.2, 6.4]	4.6 [2.7, 9.5]	5.5 [3.1, 8.3]	2.7 [2.0, 4.0]	3.0 [1.9, 6.2]
Platelets, minimum first 24 h, × 10⁹/L	153.0 [72.0, 238.5]	157.0 [97.0, 232.0]	120.5 [39.8, 203.2]	123.0 [62.0, 204.0]	147.5 [68.2, 245.0]	161.0 [76.2, 217.8]
D-dimer, first, ng/mL	3535.0 [1569.0, 7745.0]	3130.0 [1453.0, 8037.0]	3539.0 [2045.5, 7599.5]	3812.0 [2081.5, 8072.5]	3340.5 [1752.2, 6614.0]	3934.0 [1623.0, 5673.0]
CRP, first, mg/L	226.4 [155.5, 316.7]	129.4 [67.4, 216.2]	208.3 [130.6, 282.8]	135.0 [94.6, 145.9]	222.9 [156.9, 299.0]	203.1 [77.2, 241.2]
Sepsis source						
Abdominal	124 (20.3%)	186 (8.4%)	32 (19.2%)	21 (11.9%)	12 (10.7%)	6 (5.3%)
Pneumonia/respiratory	197 (32.3%)	640 (29.0%)	53 (31.7%)	64 (36.2%)	47 (42.0%)	51 (44.7%)
Urinary tract	69 (11.3%)	449 (20.4%)	13 (7.8%)	39 (22.0%)	6 (5.4%)	14 (12.3%)
Comorbidities						
Chronic kidney disease	109 (17.9%)	633 (28.7%)	29 (17.4%)	54 (30.5%)	25 (22.3%)	43 (37.7%)
Diabetes mellitus	200 (32.8%)	735 (33.3%)	46 (27.5%)	56 (31.6%)	35 (31.2%)	37 (32.5%)
Hypertension	315 (51.6%)	1381 (62.6%)	75 (44.9%)	94 (53.1%)	51 (45.5%)	67 (58.8%)
Ischemic heart disease	122 (20.0%)	886 (40.2%)	37 (22.2%)	61 (34.5%)	23 (20.5%)	44 (38.6%)

Values are shown for selected matched phenotype pairs to provide concise clinical context. The full native-cluster table, including all five Sheba phenotypes and all six MIMIC-IV phenotypes separately, is provided in Supplementary Table S1. Mortality values refer to patients alive at the 96-h landmark. Continuous variables are shown as mean ± SD or median [IQR], as appropriate. Lactate is reported in mmol/L.

After clustering and DTW-based refinement, one small cluster (*n* = 24) representing an extreme early high-dose “Fulminant Shock” pattern did not meet the prespecified minimum size and was absorbed into the most similar phenotype.

The final phenotype composition was low dose–early resolver (54.9%), high dose–early resolver (14.2%), intermediate dose–gradual wean (15.3%), high dose–slow resolver (5.7%), and intermediate dose–non-resolver (10.0%).

### Trajectory phenotypes (qualitative)

**Fig. 1:**
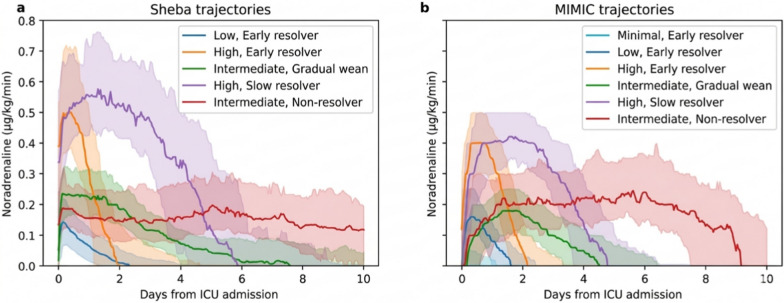
Median NA trajectories by cluster. Solid lines represent the cluster‐specific median dose (µg/kg/min) from ICU admission; shaded bands represent the interquartile range. **a** Sheba cohort trajectories, **b** MIMIC-IV cohort trajectories

Median NA trajectories (IQR ribbons) presented distinct temporal exposure patterns across clusters (Fig. 1).

In general, lower risk clusters (low dose–early resolver for Sheba, minimal dose–early resolver and low dose–early resolver for MIMIC-IV) exhibited quicker early de-escalation, lower AUCs on days 2–4, and earlier t50. Higher risk clusters (high dose–slow resolver and intermediate dose–non-resolver) maintained higher exposures into days 2–4, with longer t50 and more time above 0.10 µg/kg/min.

### Survival analyses

**Fig. 2:**
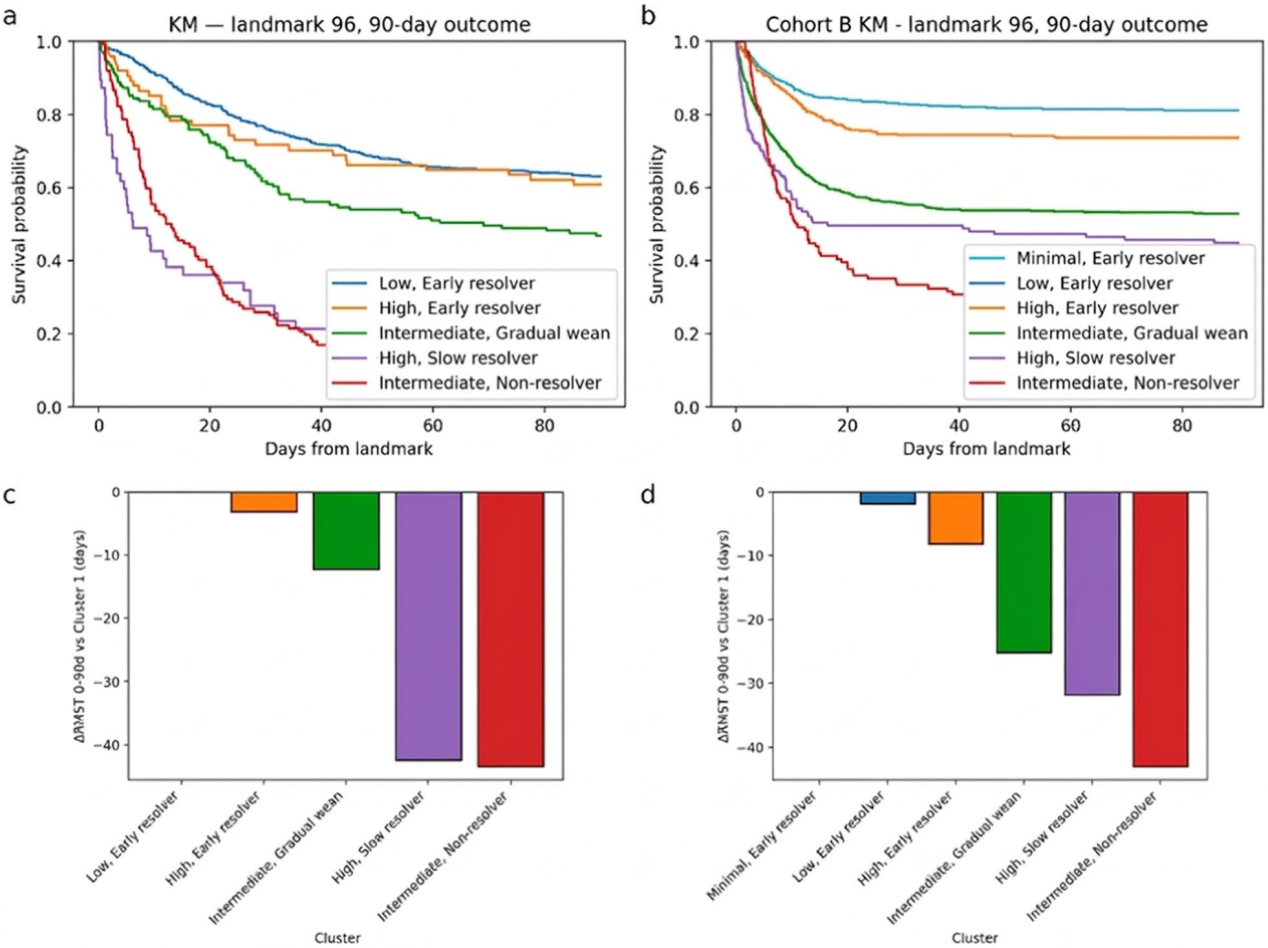
Survival by NA‐trajectory cluster and ΔRMST versus lowest mortality cluster (landmark 96 h). **a** Kaplan–Meier survival to 90 days by cluster—Sheba cohort. **b** Kaplan–Meier survival to 90 days—MIMIC-IV cohort. **c** Difference in restricted mean survival time (ΔRMST) at 90 days relative to the first, lowest mortality cluster—Sheba cohort. **d** ΔRMST at 90 days—MIMIC-IV cohort

At prespecified landmarks (e.g., 96 h), Kaplan–Meier analyses demonstrated clear and consistent separation in 90-day survival across trajectory phenotypes, with lowest mortality in the minimal/low–early resolver and highest mortality in the intermediate–non-resolver phenotype (Fig. 2). Corresponding Cox models showed progressively higher hazard ratios for higher risk phenotypes.

Similar mortality gradients were also observed at 30 days in both cohorts, supporting that the prognostic signal of the trajectory phenotypes was already evident in the earlier post-shock period (Supplementary Table S1 and Figure S5).

### Individual-feature analysis (phenotype interpretability)

**Fig. 3:**
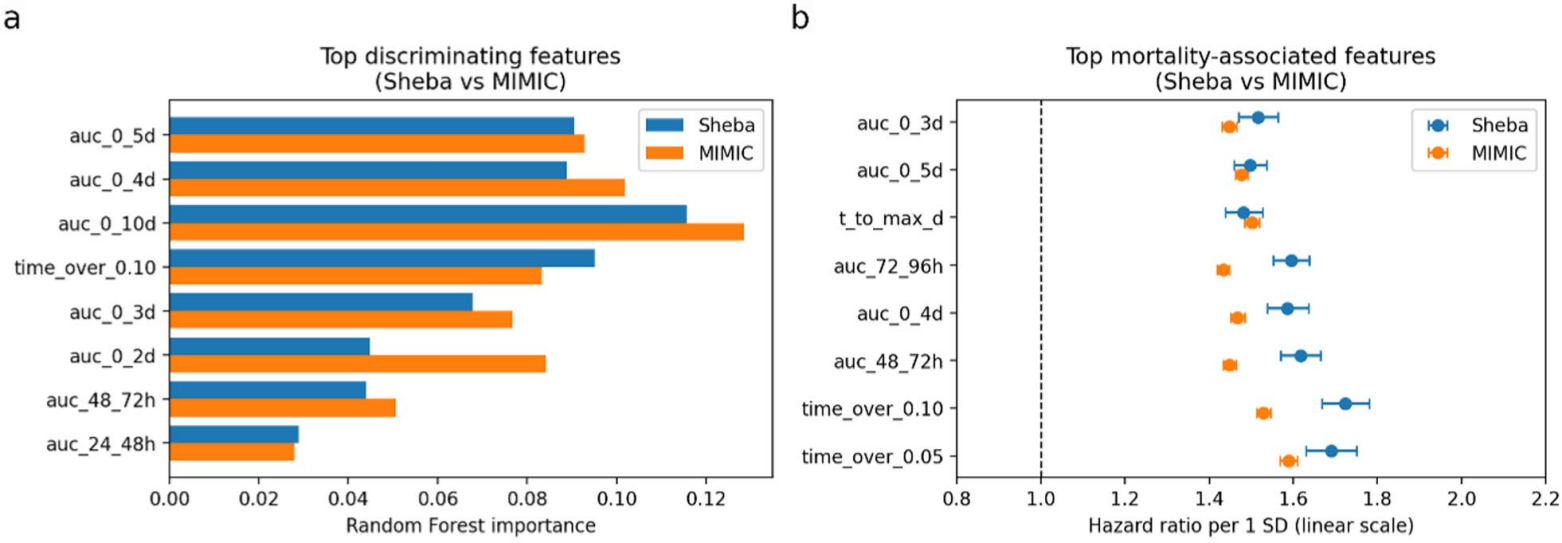
Top discriminating features and top mortality-associated features. **a** Top discriminating features of NA trajectories in Sheba and MIMIC-IV. Bar plot showing the eight highest ranking features for separating cluster phenotypes in each cohort, based on combined random forest importance and ANOVA F-statistics. Exposure-related variables, particularly cumulative AUC windows (0–2 to 0–10 days) and mid-course exposure (AUC 24–48 h and 48–72 h), consistently ranked highest in both datasets, indicating strong cross-cohort agreement on the dimensions that define trajectory phenotypes. **b** Top mortality-associated NA-trajectory features (96-h landmark, 90-day mortality). Forest plot showing hazard ratios per 1-SD increase for the eight most prognostic features shared by Sheba and MIMIC-IV, using linear HR scale. Cumulative exposure features (e.g., AUC 0–10 days, 0–5 days, 0–4 days, 0–3 days) and early mid-course exposure (AUC 48–72 h, 72–96 h) show the strongest mortality associations in both cohorts, with broadly consistent effect sizes. Vertical dashed line denotes HR = 1.0 (no association)

Across both cohorts, phenotype separation and mortality associations were dominated by exposure magnitude and persistence, particularly cumulative NA exposure and mid-course (24–72 h) dosing, as well as time-above physiologic thresholds (Fig. 3). Univariable analyses showed monotonic increases in mortality risk across prognosis-ordered phenotypes, with time to maximal NA dose emerging as a strong and consistent predictor independent of cumulative exposure. Withdrawal-speed metrics contributed comparatively little additional prognostic information.

### Consistency of covariate effects across landmarks

**Fig. 4:**
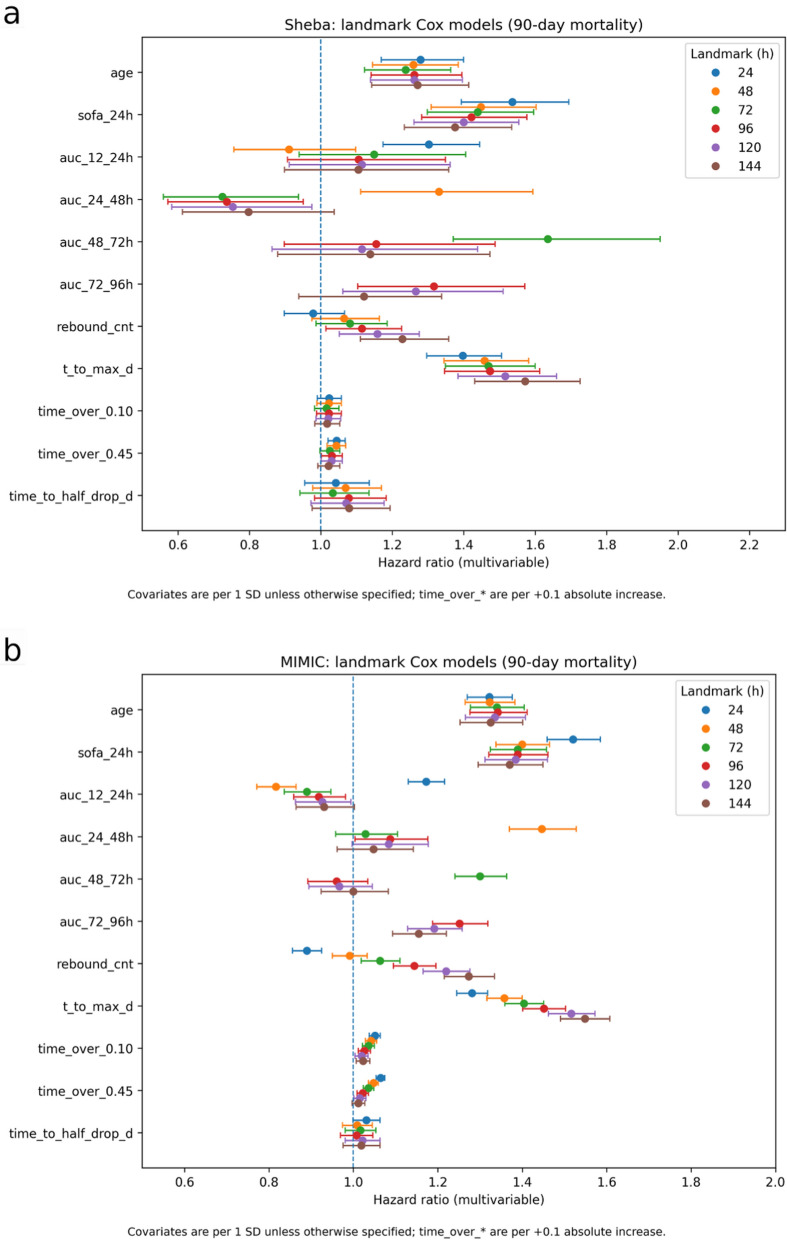
Multivariate landmark Cox models (24–144 h). Forest plots of hazard ratios (HRs) and 95% confidence intervals from multivariable landmark Cox proportional hazards models estimated at prespecified landmarks (24, 48, 72, 96, 120, and 144 h after ICU admission) in **a** the Sheba derivation cohort and **b** the external MIMIC-IV validation cohort. Points denote HR estimates and horizontal bars denote 95% confidence intervals; colors indicate landmark time. Continuous predictors were standardized to 1 SD to enable comparison of effect magnitudes; *time_over_* variables are scaled per + 0.1 absolute increase. The vertical dashed line indicates HR = 1.0. Across both cohorts, age and SOFA-24 h were consistently associated with increased mortality risk, and norepinephrine-trajectory features reflecting delayed escalation (*t_to_max_d*), rebound (*rebound_cnt*), and time above dose thresholds (*time_over_0.10*, *time_over_0.45*) showed reproducible adverse associations. Decomposition of exposure into non-overlapping AUC windows highlighted a stronger and more consistent association for later-phase burden (48–96 h) compared with earlier windows

Multivariable landmark Cox models (24–144 h) showed broadly consistent covariate–mortality associations across time points and across health systems (Fig. 4). Age and SOFA-24 h were consistently associated with higher 90-day mortality in both cohorts. At the 96-h landmark, sensitivity analyses replacing SOFA with SOFA without the cardiovascular component yielded concordant associations for both 30-day and 90-day mortality in Sheba and MIMIC-IV, in both base and extended models.

Several norepinephrine-trajectory features also demonstrated reproducible independent associations, particularly delayed escalation (time to maximal dose, *t_to_max_d*), rebound frequency (*rebound_cnt*), and persistence above clinically relevant dose thresholds (*time_over_0.10* and *time_over_0.45*). When exposure was decomposed into non-overlapping time-window AUC components, later-phase burden (days 3–4; AUC 48–72 h and 72–96 h) exhibited the most consistent adverse associations at the corresponding landmarks, whereas earlier window AUCs (day 1–2) showed weaker and less stable effects. Overall, the trajectory features that distinguish the phenotypes were also those most consistently associated with outcome, with similar directionality and magnitude in the external MIMIC-IV cohort.

### Internal validation

Prognostic performance of landmark Cox models incorporating trajectory phenotypes was assessed with and without adjustment for baseline severity. At the 96-h landmark, cluster-only models achieved a Harrell’s C-index of 0.65, increasing to 0.70–0.72 after covariate adjustment. Across landmarks (24–144 h), cluster-only C-indices ranged from 0.63 to 0.66, and adjusted models from 0.68 to 0.72 (Supplementary Fig. S4). Bootstrap optimism correction showed minimal attenuation (< 0.02).

### External validation

Generalizability was evaluated in the independent MIMIC-IV cohort using the frozen derivation pipeline and prespecified phenotype mapping. Mortality gradients were preserved across externally assigned phenotypes, reproducing the internal risk hierarchy (Fig. 2). In unrefitted external Cox models, cluster-only Harrell’s C-indices ranged from 0.61 to 0.63 across landmarks (0.61 at 96 h), with closely parallel development and validation performance (Supplementary Fig. S4).

### Early cluster prediction

#### Derivation cohort (Sheba)

Although phenotypes were defined from full 10-day NA trajectories, a phenotype-related signal was already present at 24 h and became more discriminative by 48 h. Using 24-h features, the temperature-scaled multinomial model achieved 85% accuracy; 75% of patients met the high-confidence threshold (P_max ≥ 0.80), with 94% accuracy within this subgroup. By 48 h, overall accuracy increased to 89%, high-confidence coverage to 78%, and high-confidence subgroup accuracy to 95%. Performance remained stable at later horizons, with 72-h and 96-h models achieving 90% and 85% overall accuracy, respectively, and 96% accuracy within high-confidence subsets.

### External validation cohort (MIMIC-IV)

External performance closely paralleled derivation results. Using 24-h features, the model achieved 86% accuracy; 88% of patients met the high-confidence threshold, with 91% accuracy within this subgroup. By 48 h, overall accuracy increased to 88%, high-confidence coverage to 89%, and high-confidence subgroup accuracy to 93%. Performance remained stable to improved at later horizons, reaching 89% accuracy at 72 h and 90% at 96 h, with high-confidence subgroup accuracy of 93% and 95%, respectively. Misclassification patterns mirrored those observed in Sheba, with errors concentrated among intermediate phenotypes, whereas extreme phenotypes were rarely confused with one another. Temperature scaling yielded stable probability estimates across horizons. Detailed performance metrics and confusion matrices are provided in Supplementary Tables S5 and S6.

**Fig. 5:**
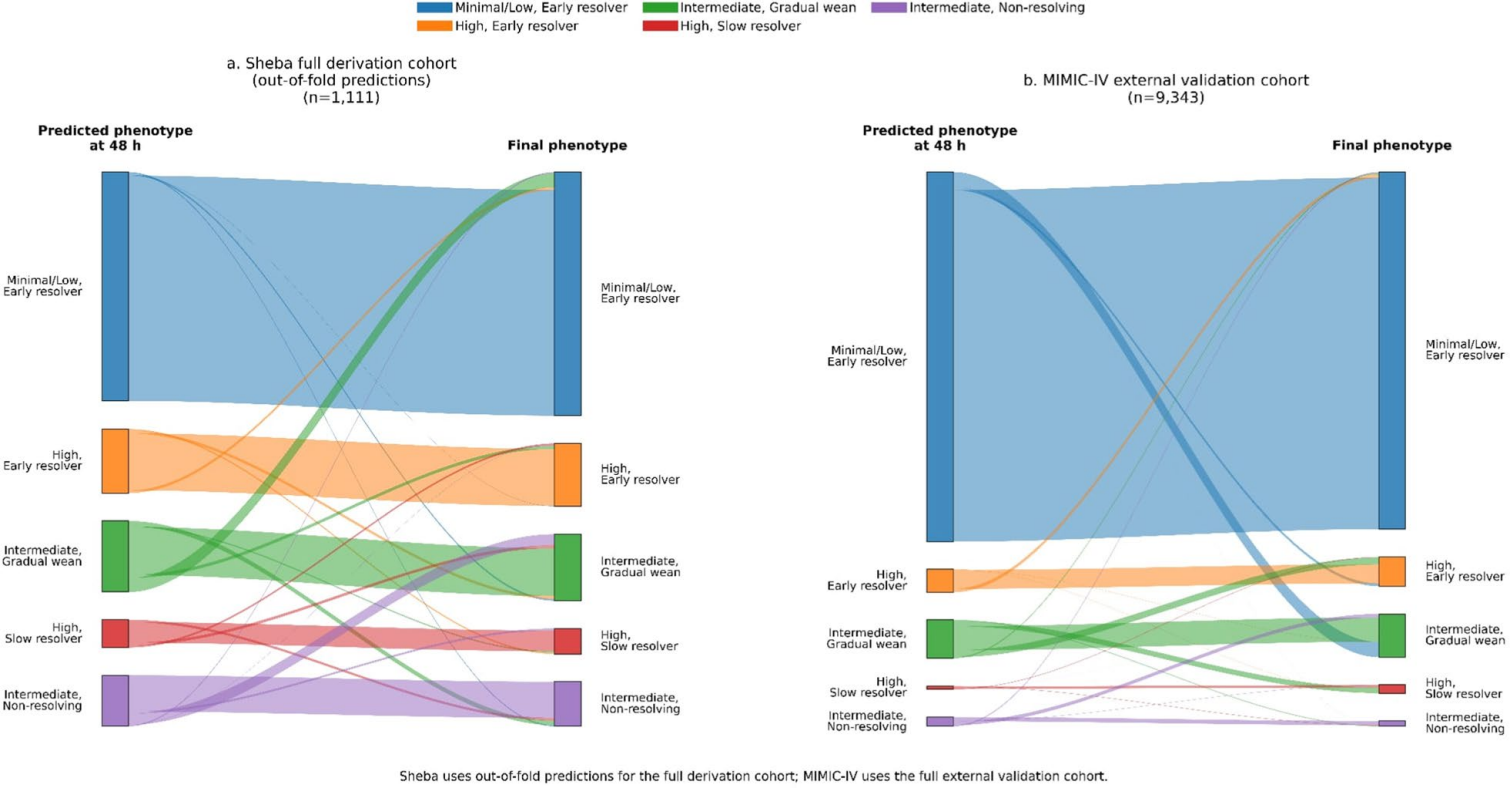
Flow from 48-h predicted phenotype to final trajectory phenotype assignment. Alluvial diagrams show the relationship between phenotype predicted from truncated 48-h norepinephrine exposure data and final phenotype assignment based on the full trajectory framework in the derivation cohort (**a**, Sheba full derivation cohort with out-of-fold predictions) and the external validation cohort (**b**, MIMIC-IV). Left-sided strata represent the phenotype predicted at 48 h; right-sided strata represent the final assigned phenotype. Ribbon widths are proportional to patient counts. Dominant straight flows indicate stable early classification, whereas crossing flows indicate directional misclassification, most prominently among intermediate phenotypes

To complement the confusion matrices, Fig. 5 provides an alluvial visualization of 48-h predicted versus final phenotype assignment, illustrating classification stability and the main directional misclassification patterns.

## Discussion

In this study, we derived and externally validated NA dose-trajectory phenotypes in septic shock using a transparent clustering framework applied independently to two large ICU populations. Across both cohorts, the approach revealed distinct and clinically interpretable NA trajectories that corresponded closely with differences in 90-day mortality, reinforcing that dynamic vasopressor response captures meaningful heterogeneity in hemodynamic response during septic shock. We used 90-day mortality as the primary endpoint and also examined 30-day mortality. Similar risk gradients at 30 days indicate that the prognostic signal was already present early after shock.

### Phenotypic structure and hemodynamic-response interpretation

The trajectory phenotypes reflected clinically recognizable hemodynamic-response patterns. Favorable groups showed rapid early de-escalation, low cumulative exposure during days 2–4, and earlier half-dose recovery (t50), patterns compatible with earlier hemodynamic stabilization and treatment responsiveness. Unfavorable groups required sustained high doses, exhibited rebound-prone behavior, or developed late dose escalation patterns likely reflecting more persistent vasoplegia, ongoing shock, or evolving multiorgan dysfunction [7, 11, 12].

An important interpretive point is that these phenotypes were designed to describe the evolution of noradrenaline requirement itself. They therefore represent hemodynamic-response phenotypes rather than pure biological endotypes of septic shock. Importantly, NA dose is not a direct physiological measurement. It reflects a composite of vascular responsiveness, disease severity, volume status, cardiac function, institutional practice, and clinician titration against conventional hemodynamic targets. Accordingly, the dose required over time should not be viewed as an arbitrary treatment choice alone, but as a treatment-response trajectory with physiological correlates that also reflects bedside management. To provide clinical and biological context for these hemodynamic-response patterns, we also examined phenotype-level distributions of lactate, platelet count, CRP, D-dimer, and infection source. Notably, the high early resolver phenotype showed marked early metabolic and hematologic derangement yet lower mortality than the high slow resolver and non-resolver phenotypes, suggesting that persistence of noradrenaline requirement conveys information beyond initial insult severity alone. Consistent with a more coagulopathic phenotype, the high slow resolver cluster showed the highest early D-dimer values in both cohorts.

Two design choices enhanced interpretability and robustness: (1) outcome-agnostic clustering with outcome-based renumbering, which prevented outcome leakage while producing clinically intuitive labels, and (2) a rich feature set that captured both smooth and abrupt changes in vasopressor support, yielding a stable and high-resolution phenotypic structure.

### Stability and cross-cohort reproducibility of the cluster structure

The core trajectory families were highly reproducible across the two health systems, with only two expected differences at the extremes of severity and duration.

1. Cluster number differed slightly.
  Sheba ultimately produced five stable clusters after the very small “Fulminant Shock’’ group—marked by extreme early NA requirements and very high early mortality—was absorbed during DTW refinement. This reflected a deliberate preference for a stable and reproducible phenotype structure over retention of very small extreme-tail groups as standalone clusters. While such rare micro-phenotypes may still be clinically informative, we considered them less suitable as primary phenotypes unless they met the prespecified minimum-size criterion. In contrast, MIMIC-IV retained six stable clusters.
2. Prolonged-exposure phenotypes differed in duration.
  Sustained and late-escalating phenotypes (e.g., “Intermediate-dose Gradual Wean’’ and “Intermediate-dose Non-resolver”) persisted longer in Sheba, even though their shapes were nearly identical to their counterparts in MIMIC-IV.

These discrepancies likely reflect institutional differences in aggressive support and end-of-life practices. Sheba commonly maintains full hemodynamic support for longer periods, generating both the brief, high-dose ‘Fulminant Shock’ trajectory and prolonged vasopressor courses in survivors. At the MIMIC-IV source hospital, earlier transition to comfort-focused care and a pharmacy/protocol norepinephrine ceiling of 0.5 µg/kg/min may have limited representation of very high dose exposures and shortened observable trajectories, thereby influencing the final cluster structure.

Despite these differences in duration, the shape, relative structure, and prognostic ordering of the phenotypes were strongly preserved across cohorts, supporting that trajectory-based phenotyping captures stable and transportable dimensions of vasopressor-response behavior in septic shock.

### Drivers of phenotypic separation and mortality

Individual-feature analyses showed that dose duration and persistence metrics were the primary determinants of both phenotype separation and mortality. Cumulative AUC windows, mid-course exposure (24–72 h), and time-over-threshold measures consistently ranked highest across cohorts [3–5]. This finding should be interpreted in light of the prespecified feature representation: the clustering library intentionally included overlapping cumulative AUC windows and time-over-threshold metrics to capture clinically interpretable dimensions of vasopressor burden and persistence. Therefore, the emergence of exposure-persistence phenotypes is partly a consequence of this predefined representation rather than a representation-neutral discovery.

However, the phenotypes were not determined by cumulative exposure alone. Time to maximal NA dose (t_to_max_d) emerged as a strong, reproducible predictor in both Sheba and MIMIC-IV, indicating that the timing of the hemodynamic peak carries adverse prognostic information beyond total exposure burden [6]. This may reflect ongoing shock physiology, delayed hemodynamic stabilization, incomplete control of the underlying insult, or progression of multiorgan dysfunction.

Withdrawal-speed features, including early slopes, t50, and half-drop metrics, added comparatively little incremental prognostic value. This suggests that the dominant independent signal lies less in the fine structure of vasopressor tapering and more in the broader pattern of dose burden, escalation timing, persistence, and failure of resolution.

Multivariable landmark models (24–144 h) showed stable effect directions across time and across cohorts, with expected attenuation and occasional CI overlap due to collinearity and smaller samples at later horizons (Fig. 4).

These findings should be interpreted as both confirmatory and additive. The broad clinical observation that persistent or escalating vasopressor requirement is associated with worse outcome is already well recognized. The added value of the present framework is that it extends this intuition beyond dose or duration alone by integrating multiple trajectory descriptors, including cumulative exposure, threshold persistence, timing of maximal dose, rebound behavior, and recovery metrics. Thus, the framework does not merely restate that prolonged vasopressor dependence is harmful; it converts clinically recognizable vasopressor-response patterns into a transparent, externally tested structure for dynamic risk stratification, cohort enrichment, and future trajectory-informed studies.

Taken together, these results support interpretation of the clusters as clinically interpretable hemodynamic-response trajectories, while acknowledging that alternative representations, such as de-correlated feature sets, raw time-series clustering alone, or multimodal physiologic inputs, might yield different phenotype structures.

### Internal and external prognostic validation

In the derivation cohort, trajectory membership demonstrated strong prognostic separation at 96 h (optimism-corrected Harrell’s *C* = 0.636). The entire frozen pipeline applied to MIMIC-IV reproduced the trajectory families and demonstrated monotonic 90-day mortality gradients from lowest to highest risk clusters (16–69%), which is highly comparable to the prognostic range captured by complex biological subphenotypes [13, 14].

When cluster number was treated as an ordinal variable in a non-refitted Cox model, discrimination in MIMIC-IV remained meaningful (C-index 0.619; AUROC 0.63), consistent with expected attenuation when transporting dynamic physiologic models across systems.

Feature-outcome associations were directionally and rank-order consistent across cohorts, further supporting external validity and physiologic transportability.

### Early recognition of phenotypes

The ability to identify NA trajectory phenotypes from data available within the first 24 h, with stronger discrimination by 48 h, indicates that meaningful differences in hemodynamic trajectories emerge early in the course of illness. The reproducibility of this early signal across two health systems—despite differences in documentation density, patient mix, and clinical workflows—suggests that the trajectory structure reflects stable and transportable patterns embedded in routine dosing data rather than artifacts of local practice.

Early misclassification patterns were clinically coherent and consistent with the expected temporal evolution of the phenotypes. In the derivation cohort, the primary source of ambiguity involved the intermediate gradual wean and minimal/low early resolver groups, which share a low-dose pattern during the first 24–48 h before diverging thereafter. This early similarity accounts for the directional misclassification of those clusters and reflects the genuine difficulty of distinguishing these two trajectories before mid-course exposure accumulates. In contrast, the high slow resolver phenotype begins at substantially higher doses and remained well separated in the derivation data; its occasional reassignment to intermediate, gradual wean cluster in the external cohort likely reflects differences in early exposure distributions and the limited discriminatory power of the truncated early-feature set, rather than true overlap in the underlying trajectory pattern.

Although the classifier demonstrated strong generalizability, the external misclassification patterns underscore that site-level calibration may be necessary to optimize early prediction. Differences in dose ceilings, recording density, and the distribution of early exposures can shift the mapping between features and phenotypes, and modest recalibration may further improve discrimination, particularly for intermediate phenotypes.

Importantly, 24-h and 48-h classification should not be considered clinically equivalent: a phenotype-related signal was already present at 24 h, but classification was more stable and informative by 48 h.

Together, these findings show that the core differences encoded within NA trajectories arise early and are reproducible across health systems. However, the present study demonstrates early descriptive and prognostic classification rather than an established phenotype-specific treatment algorithm. The intended near-term use of early classification is therefore best viewed in three domains: dynamic risk stratification, enrichment of future observational or interventional studies, and generation of hypotheses regarding trajectory-specific supportive strategies.

A conceptual clinical interpretation is that a high-confidence assignment at 24 h may provide early risk awareness, whereas by 48 h, when phenotype discrimination was stronger and misclassification decreased, persistence of a slow-resolving or non-resolving pattern may identify a more prognostically enriched subgroup for closer reassessment or for inclusion in future trials testing monitoring or de-escalation strategies. Conversely, early-resolving patterns may help define lower risk comparator groups in research settings. At this stage, these interpretations should be viewed as hypothesis-generating; prospective studies are required to determine whether early phenotype assignment can meaningfully influence management or patient outcomes.

In future prospective work, this framework could be evaluated as a decision-support layer that continuously updates phenotype probabilities from routinely charted norepinephrine exposure during the first 24–48 h. Rather than dictating a fixed treatment algorithm, such a tool could flag patients whose early course appears more compatible with slow resolution or non-resolution, thereby prompting closer reassessment of hemodynamic trajectory, supportive intensity, and readiness for de-escalation. Because calibration differed across cohorts, any such implementation would require local recalibration before bedside deployment.

### Limitations

This study has several limitations. First, its retrospective design precludes causal inference, and NA dosing inevitably reflects both underlying illness severity and clinician decision-making (“confounding by indication”). Second, although trajectory phenotypes were reproducible across two large health systems, differences in treatment-limitation practices, including withholding or withdrawal of life-sustaining therapy, and differences in duration of supportive care may have influenced the expression of prolonged trajectories. Third, despite extensive feature engineering, unmeasured physiologic variables—such as microcirculatory flow, cardiac output, or autonomic tone—may contribute to phenotype structure but were not directly captured. Fourth, dose trajectories were reconstructed from routine medication administration records, and varying documentation density across systems may introduce subtle measurement error. Finally, while early classification performance generalized well, calibration differed between cohorts, indicating that site-specific recalibration procedures may be necessary to optimize early phenotype assignment in new clinical environments.

## Conclusions

Noradrenaline-trajectory phenotypes derived from routine medication data were clinically interpretable, prognostically coherent, and reproducible across two ICU populations. Despite minor cross-system differences, the core trajectory shapes and associated mortality gradients were conserved. Two exposure-persistence features—time to maximal NA dose and cumulative NA load during days 2–4—consistently dominated prognosis and drove phenotype separation. Because these defining elements were already detectable at 24 h and became more discriminative by 48 h, trajectory phenotypes may support dynamic risk stratification after initial resuscitation and phenotype-enriched clinical trial design. Their role in guiding monitoring, de-escalation, or supportive strategies remains hypothesis-generating and requires prospective evaluation.

## Supplementary Information

Additional file1 (DOCX 620 kb)

## Data Availability

The datasets used and/or analyzed during the current study are available from the corresponding author upon reasonable request.
