# Supplementary material for: Noradrenaline-trajectory phenotypes in septic shock: derivation and external validation in two independent cohorts
Source: Intensive Care Med Exp. 2026 Jun 22;14:77. doi: 10.1186/s40635-026-00910-8 (PMC13287178; doi:10.1186/s40635-026-00910-8)
Supplement: Supplementary file 1 — Additional file1 (DOCX 620 kb) [file 40635_2026_910_MOESM1_ESM.docx]

**Supplement**

This Supplement provides additional methodological detail, full phenotype characteristics, feature definitions, clustering diagnostics, early-prediction performance metrics, and external validation results. It is structured as follows:

1. Cohort derivation and harmonization across cohorts (Figure S1).
2. Phenotype characteristics (Table S1),
3. Feature engineering (Table S2),
4. Clustering process and K selection (Figure S2, Table S3),
5. DTW refinement and absorption behavior (Figure S3),
6. Survival model validation (Figure S4),
7. Sensitivity analysis of feature-Set Size for early phenotype prediction,
8. Early prediction results (Tables S5, S6a-d),
9. Multivariable Cox analyses methods.

**Cohort derivation and harmonization across databases**

The Sheba derivation cohort was assembled using a stepwise clinical definition of septic shock among adult ICU admissions: local clinician-documented septic shock diagnosis, biochemical support with lactate >2 mmol/L measured prior to or at ICU admission, and evidence of active norepinephrine infusion around ICU admission. A documented patient weight was additionally required because Sheba norepinephrine doses were standardized to µg/kg/min, necessitating weight-based conversion; this resulted in exclusion of 4 admissions with missing weight. The MIMIC-IV external validation cohort was derived from the full set of ICU stays in MIMIC-IV v2.2 using the published Sepsis-3 computable phenotype (mimiciv_derived.septic_shock) as the septic shock ascertainment step, after which we required documented norepinephrine/noradrenaline infusion records in ICU inputevents to enable trajectory reconstruction. Unlike Sheba, an explicit weight-availability filter was not required for MIMIC-IV in the exported cohort file because norepinephrine infusion rates in inputevents are frequently recorded directly in weight-normalized units (e.g., µg/kg/min), allowing construction of standardized exposure trajectories without a separate weight field. Together, these flow diagrams summarize cohort selection and highlight that differences in inclusion steps primarily reflect cross-system documentation structure rather than substantive differences in clinical intent.

**Figure S1. Derivation of the Sheba and MIMIC-IV cohorts for norepinephrine trajectory phenotyping in septic shock**


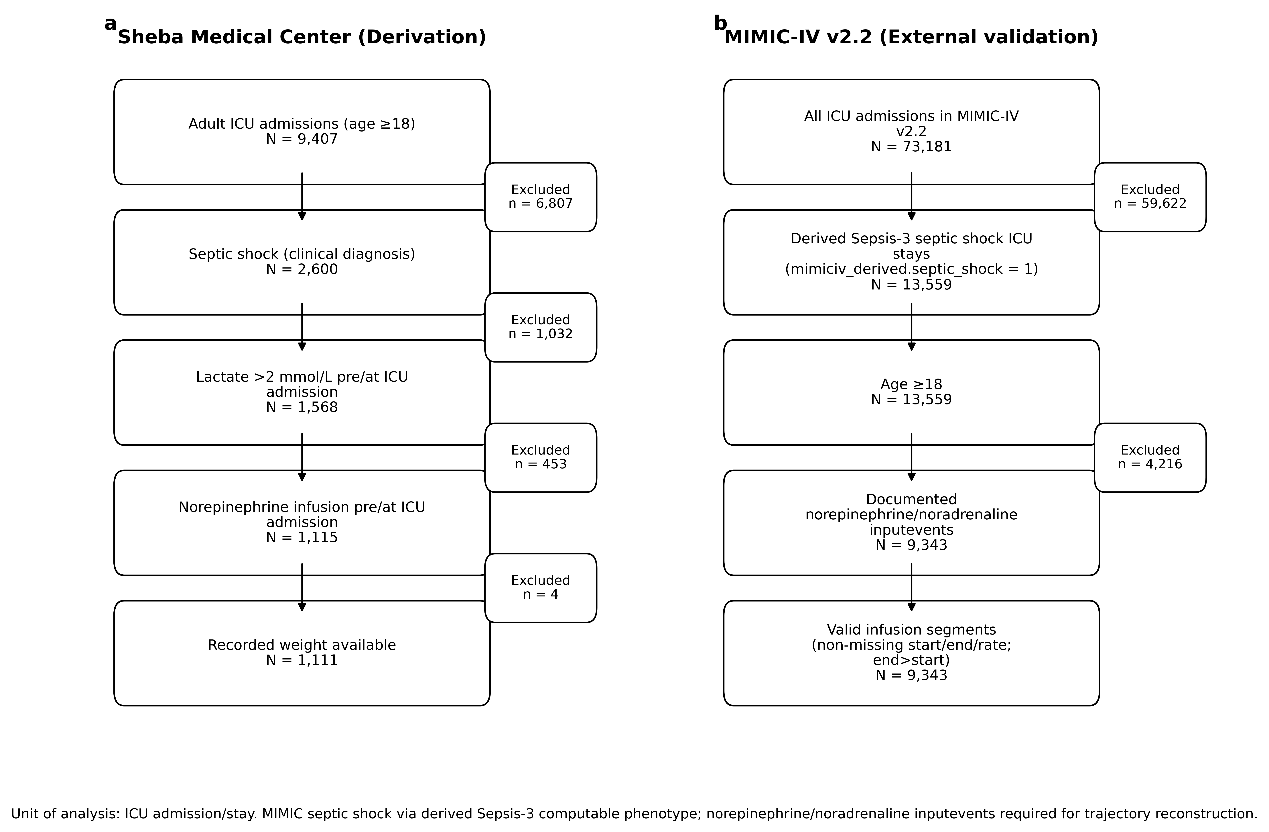


**Table S1-Supplement Table 1. Detailed demographic, severity, comorbidity, and admission profiles for all trajectory phenotypes in the Sheba derivation cohort and MIMIC-IV validation cohort**

| Variable | **Sheba Low, Early resolver (n=610)** | **MIMIC Minimal, Early resolver (n=5317)** | **MIMIC Low, Early resolver (n=2205)** | **Sheba High, Early resolver (n=158)** | **MIMIC High, Early resolver (n=912)** | **Sheba Intermediate, Gradual wean (n=167)** | **MIMIC Intermediate, Gradual wean (n=177)** | **Sheba High, Slow resolver (n=64)** | **MIMIC High, Slow resolver (n=618)** | **Sheba Intermediate, Non-resolver (n=112)** | **MIMIC Intermediate, Non-resolver (n=114)** |
| --- | --- | --- | --- | --- | --- | --- | --- | --- | --- | --- | --- |
| **Demographics, severity & mortality** | | | | | | | | | | | |
| Age, years | 63.0 ± 15.3 | 66.9 ± 15.2 | 67.3 ± 15.1 | 63.3 ± 15.3 | 65.1 ± 14.6 | 61.1 ± 14.6 | 62.6 ± 13.8 | 62.0 ± 14.8 | 65.5 ± 15.4 | 62.5 ± 13.6 | 63.7 ± 15.0 |
| Male sex | 344 (56.4%) | 3092 (58.2%) | 1287 (58.4%) | 105 (66.5%) | 559 (61.3%) | 100 (59.9%) | 106 (59.9%) | 41 (64.1%) | 364 (58.9%) | 72 (64.3%) | 69 (60.5%) |
| APACHE II | 29.3 ± 8.8 | 25.7 ± 8.4 | 29.9 ± 8.2 | 33.4 ± 8.7 | 30.6 ± 7.8 | 31.5 ± 7.1 | 34.6 ± 7.4 | 31.2 ± 8.2 | 34.1 ± 8.0 | 30.1 ± 6.5 | 29.1 ± 9.0 |
| SOFA (24h max) | 12.5 ± 3.9 | 9.5 ± 3.5 | 11.1 ± 3.5 | 15.9 ± 3.8 | 11.7 ± 3.6 | 14.9 ± 3.6 | 13.0 ± 2.9 | 15.9 ± 3.5 | 12.5 ± 3.5 | 14.1 ± 3.6 | 11.7 ± 3.8 |
| SOFAwithoutCardio (24h) | 9.7 ± 3.5 | 6.6 ± 3.4 | 8.2 ± 3.4 | 12.4 ± 3.6 | 9.0 ± 3.4 | 11.3 ± 3.5 | 10.1 ± 2.8 | 12.4 ± 3.3 | 9.7 ± 3.2 | 10.8 ± 3.2 | 9.4 ± 3.5 |
| 30 day mortality (96h landmark) | 23.11% | 15.16% | 17.07% | 32.05% | 44.55% | 39.58% | 50.39% | 73.47% | 25.63% | 74.11% | 66.67% |
| 90 day mortality (96h landmark) | 36.97% | 16.38% | 18.68% | 42.31% | 47.17% | 53.47% | 55.12% | 83.67% | 26.46% | 89.29% | 69.30% |
| Acute respiratory failure | 127 (20.8%) | 2233 (42.0%) | 1177 (53.4%) | 42 (26.6%) | 558 (61.2%) | 42 (25.1%) | 106 (59.9%) | 13 (20.3%) | 392 (63.4%) | 29 (25.9%) | 73 (64.0%) |
| Mechanical ventilation (admission) | 495 (81.1%) | 3495 (65.7%) | 1731 (78.5%) | 140 (88.6%) | 771 (84.5%) | 146 (87.4%) | 156 (88.1%) | 58 (90.6%) | 548 (88.7%) | 107 (95.5%) | 88 (77.2%) |
| Acute kidney injury (admission) | 298 (48.9%) | 3026 (56.9%) | 1403 (63.6%) | 76 (48.1%) | 655 (71.8%) | 93 (55.7%) | 150 (84.7%) | 33 (51.6%) | 467 (75.6%) | 54 (48.2%) | 83 (72.8%) |
| Cardiogenic shock | 17 (2.8%) | 543 (10.2%) | 305 (13.8%) | 8 (5.1%) | 190 (20.8%) | 9 (5.4%) | 41 (23.2%) | 5 (7.8%) | 115 (18.6%) | 9 (8.0%) | 30 (26.3%) |
| RRT any ICU | 164 (27.4%) | 699 (13.1%) | 405 (18.4%) | 86 (54.4%) | 345 (37.8%) | 99 (59.3%) | 101 (57.1%) | 47 (73.4%) | 213 (34.5%) | 67 (59.8%) | 73 (64.0%) |
| Adrenaline any ICU | 99 (16.5%) | 620 (11.7%) | 382 (17.3%) | 77 (48.7%) | 178 (19.5%) | 54 (32.3%) | 80 (45.2%) | 41 (64.1%) | 220 (35.6%) | 38 (33.9%) | 35 (30.7%) |
| Vasopressin any ICU | 412 (68.8%) | 1055 (19.8%) | 1087 (49.3%) | 153 (96.8%) | 632 (69.3%) | 161 (96.4%) | 166 (93.8%) | 64 (100.0%) | 512 (82.8%) | 110 (98.2%) | 92 (80.7%) |
| Milrinone any ICU | 56 (9.3%) | 263 (4.9%) | 82 (3.7%) | 24 (15.2%) | 82 (9.0%) | 39 (23.4%) | 9 (5.1%) | 21 (32.8%) | 15 (2.4%) | 26 (23.2%) | 8 (7.0%) |
| Dobutamine any ICU | 16 (2.7%) | 194 (3.6%) | 148 (6.7%) | 7 (4.4%) | 90 (9.9%) | 7 (4.2%) | 28 (15.8%) | 3 (4.7%) | 69 (11.2%) | 5 (4.5%) | 25 (21.9%) |
| Dopamine any ICU | 81 (13.5%) | 319 (6.0%) | 164 (7.4%) | 30 (19.0%) | 105 (11.5%) | 44 (26.3%) | 39 (22.0%) | 23 (35.9%) | 84 (13.6%) | 30 (26.8%) | 12 (10.5%) |
| Any non-NE vasopressor any ICU | 434 (72.5%) | 1549 (29.1%) | 1234 (56.0%) | 156 (98.7%) | 679 (74.5%) | 163 (97.6%) | 171 (96.6%) | 64 (100.0%) | 539 (87.2%) | 110 (98.2%) | 96 (84.2%) |
| Any inotrope any ICU | 140 (23.4%) | 825 (15.5%) | 495 (22.4%) | 88 (55.7%) | 244 (26.8%) | 77 (46.1%) | 99 (55.9%) | 49 (76.6%) | 265 (42.9%) | 49 (43.8%) | 49 (43.0%) |
| **Sepsis source** | | | | | | | | | | | |
| Sepsis source: abdominal | 124 (20.3%) | 294 (5.5%) | 186 (8.4%) | 24 (15.2%) | 74 (8.1%) | 32 (19.2%) | 21 (11.9%) | 5 (7.8%) | 64 (10.4%) | 12 (10.7%) | 6 (5.3%) |
| Sepsis source: bloodstream / line | 56 (9.2%) | 1496 (28.1%) | 637 (28.9%) | 14 (8.9%) | 277 (30.4%) | 19 (11.4%) | 68 (38.4%) | 7 (10.9%) | 225 (36.4%) | 6 (5.4%) | 39 (34.2%) |
| Sepsis source: CNS | 7 (1.1%) | 51 (1.0%) | 12 (0.5%) | 3 (1.9%) | 10 (1.1%) | 1 (0.6%) | 1 (0.6%) | 0 (0.0%) | 3 (0.5%) | 2 (1.8%) | 0 (0.0%) |
| Sepsis source: pneumonia / respiratory | 197 (32.3%) | 1508 (28.4%) | 640 (29.0%) | 48 (30.4%) | 324 (35.5%) | 53 (31.7%) | 64 (36.2%) | 16 (25.0%) | 198 (32.0%) | 47 (42.0%) | 51 (44.7%) |
| Sepsis source: skin / soft tissue | 104 (17.0%) | 296 (5.6%) | 127 (5.8%) | 17 (10.8%) | 44 (4.8%) | 22 (13.2%) | 7 (4.0%) | 3 (4.7%) | 26 (4.2%) | 11 (9.8%) | 3 (2.6%) |
| Sepsis source: urinary tract | 69 (11.3%) | 1121 (21.1%) | 449 (20.4%) | 12 (7.6%) | 154 (16.9%) | 13 (7.8%) | 39 (22.0%) | 4 (6.2%) | 130 (21.0%) | 6 (5.4%) | 14 (12.3%) |
| **Biomarkers** | | | | | | | | | | | |
| Lactate, first | 2.1 [1.4, 3.6] | 2.1 [1.4, 3.3] | 2.7 [1.8, 4.6] | 5.1 [2.5, 9.7] | 2.5 [1.6, 4.2] | 3.2 [1.9, 5.4] | 3.3 [2.4, 5.8] | 3.9 [2.4, 6.2] | 3.9 [2.4, 6.6] | 1.9 [1.3, 2.6] | 2.5 [1.7, 3.9] |
| Lactate, peak first 24h | 3.0 [2.0, 5.16] | 2.5 [1.6, 4.2] | 3.4 [2.2, 6.4] | 10.2 [4.6, 17.75] | 3.4 [2.0, 6.0] | 4.6 [2.7, 9.5] | 5.5 [3.1, 8.3] | 7.7 [4.2, 11.7] | 5.8 [3.5, 9.8] | 2.7 [2.0, 4.0] | 3.0 [1.9, 6.2] |
| Platelets, first | 180.0 [105.0, 270.5] | 176.0 [117.0, 251.0] | 184.0 [120.0, 273.0] | 119.0 [51.2, 211.5] | 163.0 [99.0, 253.0] | 151.0 [80.5, 226.5] | 165.0 [94.0, 254.0] | 78.0 [39.5, 193.2] | 189.0 [115.0, 266.0] | 167.5 [85.0, 265.2] | 170.5 [88.2, 248.0] |
| Platelets, minimum first 24h | 153.0 [72.0, 238.5] | 153.0 [102.0, 225.0] | 157.0 [97.0, 232.0] | 56.0 [14.2, 158.0] | 141.0 [77.2, 218.8] | 120.5 [39.8, 203.2] | 123.0 [62.0, 204.0] | 46.0 [15.0, 147.5] | 139.0 [80.0, 218.2] | 147.5 [68.2, 245.0] | 161.0 [76.2, 217.8] |
| CRP, first | 226.4 [155.5, 316.7] | 126.2 [65.6, 201.0] | 129.4 [67.4, 216.2] | 219.1 [142.8, 304.7] | 159.9 [73.2, 228.9] | 208.3 [130.6, 282.8] | 135.0 [94.6, 145.9] | 234.4 [133.3, 345.8] | 125.2 [71.9, 184.8] | 222.9 [156.9, 299.0] | 203.1 [77.2, 241.2] |
| D-dimer, first | 3535.0 [1569.0, 7745.0] | 3177.0 [1236.0, 6259.0] | 3130.0 [1453.0, 8037.0] | 4587.0 [1918.5, 8942.5] | 3101.5 [1351.5, 6791.0] | 3539.0 [2045.5, 7599.5] | 3812.0 [2081.5, 8072.5] | 6094.0 [2049.0, 8205.0] | 6325.0 [2426.0, 9768.5] | 3340.5 [1752.2, 6614.0] | 3934.0 [1623.0, 5673.0] |
| **Comorbidities** | | | | | | | | | | | |
| Atrial fibrillation | 106 (17.4%) | 2050 (38.6%) | 812 (36.8%) | 28 (17.7%) | 344 (37.7%) | 36 (21.6%) | 72 (40.7%) | 10 (15.6%) | 200 (32.4%) | 24 (21.4%) | 48 (42.1%) |
| Chronic kidney disease | 109 (17.9%) | 1454 (27.3%) | 633 (28.7%) | 26 (16.5%) | 292 (32.0%) | 29 (17.4%) | 54 (30.5%) | 12 (18.8%) | 154 (24.9%) | 25 (22.3%) | 43 (37.7%) |
| COPD | 66 (10.8%) | 861 (16.2%) | 378 (17.1%) | 22 (13.9%) | 149 (16.3%) | 13 (7.8%) | 29 (16.4%) | 6 (9.4%) | 111 (18.0%) | 15 (13.4%) | 20 (17.5%) |
| Diabetes mellitus | 200 (32.8%) | 1858 (34.9%) | 735 (33.3%) | 42 (26.6%) | 302 (33.1%) | 46 (27.5%) | 56 (31.6%) | 14 (21.9%) | 202 (32.7%) | 35 (31.2%) | 37 (32.5%) |
| Hyperlipidemia | 247 (40.5%) | 2201 (41.4%) | 841 (38.1%) | 53 (33.5%) | 333 (36.5%) | 62 (37.1%) | 61 (34.5%) | 26 (40.6%) | 198 (32.0%) | 46 (41.1%) | 36 (31.6%) |
| Hypertension | 315 (51.6%) | 3417 (64.3%) | 1381 (62.6%) | 70 (44.3%) | 561 (61.5%) | 75 (44.9%) | 94 (53.1%) | 24 (37.5%) | 364 (58.9%) | 51 (45.5%) | 67 (58.8%) |
| Ischemic heart disease | 122 (20.0%) | 2182 (41.0%) | 886 (40.2%) | 29 (18.4%) | 386 (42.3%) | 37 (22.2%) | 61 (34.5%) | 17 (26.6%) | 214 (34.6%) | 23 (20.5%) | 44 (38.6%) |
| Ischemic cerebrovascular event / CVA | 171 (28.0%) | 364 (6.8%) | 159 (7.2%) | 44 (27.8%) | 83 (9.1%) | 50 (29.9%) | 18 (10.2%) | 20 (31.2%) | 36 (5.8%) | 31 (27.7%) | 9 (7.9%) |
| Obesity | 80 (13.1%) | 666 (12.5%) | 256 (11.6%) | 12 (7.6%) | 125 (13.7%) | 16 (9.6%) | 31 (17.5%) | 5 (7.8%) | 76 (12.3%) | 8 (7.1%) | 18 (15.8%) |

The table presents cluster characteristics without forcing structural equivalence- Sheba: 5 native phenotypes; MIMIC-IV: 6 native phenotypes.
Because clustering was performed independently in Sheba and MIMIC-IV using the same pipeline but without forcing structural equivalence, phenotypes are comparable in their trajectory logic but not expected to be identical in baseline composition. Differences in coding practices, admission patterns, comorbidity prevalence, and illness severity across the two health systems naturally result in variations in demographic and clinical distributions within each phenotype.

These phenotype-level descriptive data provide biologic context for the trajectory phenotypes without altering the prespecified vasopressor-centered clustering framework. Several patterns are noteworthy. The High Early Resolver phenotype showed the greatest early lactate and platelet derangement, yet mortality remained lower than in the High Slow Resolver and Non-resolving phenotypes, suggesting that persistence of vasopressor requirement carries information beyond initial insult severity alone. By contrast, the High Slow Resolver phenotype showed a stronger coagulopathic profile, including the highest early D-dimer values in both cohorts, whereas the Non-resolving phenotype had very high mortality despite less extreme early lactate disturbance and was relatively enriched for respiratory sepsis. These observations support interpretation of the clusters as clinically meaningful hemodynamic-response phenotypes with biologic context, rather than arbitrary treatment behavior alone.

**Feature library and time-window engineering**

We derived a comprehensive library of noradrenaline (NE) exposure features designed to capture the magnitude, persistence, escalation kinetics, and post-peak recovery behavior of vasopressor therapy during septic shock. All features were computed from reconstructed hourly NE dose trajectories, which were generated by stitching together dose–time intervals extracted directly from the raw infusion records. Infusion rates were normalized to norepinephrine base-equivalent µg/kg/min based on the documented rate units and patient weight; overlapping infusions were combined by taking the maximal instantaneous rate. This formulation was chosen to harmonize dosing across health systems, because commercial norepinephrine preparations may be supplied as salts (for example, bitartrate/tartrate) while being labeled or reported in base-equivalent terms.

Hourly trajectories were smoothed using the user-selectable method (median, moving average, or Savitzky–Golay), and for each patient the following features were computed exactly as implemented in the application:

**Exposure magnitude (AUC).** We calculated area-under-the-curve values over fixed windows: 0–12 h, 12–24 h, 24–48 h, 48–72 h, and 72–96 h. Cumulative AUCs were computed from 0–48 h (0–2 d), 0–72 h (0–3 d), 0–96 h (0–4 d), 0–120 h (0–5 d), and 0–10 days.

**Dose intensity and escalation.** Dose intensity was characterized by the maximum NE rate achieved and the time to reach this peak. Post-peak recovery was quantified using t50, defined as the number of days from the peak to a 50% decline in dose; binary indicators of ≥50% reduction by days 2–5 were also recorded.

**Slopes and slope-angles.** Signed linear slopes were computed over 0–12 h, 12–48 h, 24–48 h, and 48–72 h using centered ordinary-least-squares regression. Slope angles (in degrees) were derived by converting slopes to per-hour scale and applying arctangent transformation; this compresses extreme values and emphasizes physiologic directionality. Angle-change metrics between early and mid windows (Δθ{0–12→24–48} and Δθ{12–48→48–72}) captured turning-point behavior.

**Threshold-based burden metrics.** Persistence above clinically relevant thresholds was quantified using global time-over-0.05 µg/kg/min, time-over-0.10 µg/kg/min, and a higher threshold time-over-0.45 µg/kg/min. In addition, window-specific time-over-0.45 metrics were calculated for 0–24 h, 24–48 h, 48–72 h, and 72–96 h. The app uses **0.45 µg/kg/min** as the high-dose threshold (not 0.50), which avoids bias introduced by MIMIC-IV’s protocolized syringe-pump truncation at 0.50 µg/kg/min.

**Rebound dynamics.** Rebounds were defined as clinically meaningful post-peak re-escalations in NE dose. To distinguish genuine re-escalations from bedside titration noise, each trajectory was interpolated and processed using two independent detectors:

1. **Strict turning-point detector**: required a decline–valley–rise structure meeting minimum absolute or relative drop/rise criteria, peak/valley prominence, and minimum time separation.
2. **Loose detector**: used relaxed prominence and gap thresholds to detect broader re-escalation phenomena.

For each patient, the final rebound count was the **maximum** of strict and loose detectors, ensuring sensitivity while suppressing noise.

All features used in clustering are provided in Supplement Table S2.

**Supplement Table S2. Noradrenaline Feature Library Used for Clustering**

**Exposure Magnitude (AUC Features)**

| **Domain** | **Feature Name** | **Description** |
| --- | --- | --- |
| Exposure Magnitude | **auc_0_12h** | AUC from 0–12 hours |
|  | **auc_12_24h** | AUC from 12–24 hours |
|  | **auc_24_48h** | AUC from 24–48 hours |
|  | **auc_48_72h** | AUC from 48–72 hours |
|  | **auc_72_96h** | AUC from 72–96 hours |
|  | **auc_0_2d** | Cumulative AUC from 0–48 hours |
|  | **auc_0_3d** | Cumulative AUC from 0–72 hours |
|  | **auc_0_4d** | Cumulative AUC from 0–96 hours |
|  | **auc_0_5d** | Cumulative AUC from 0–120 hours |
|  | **auc_0_10d** | Cumulative AUC from 0–10 days |

**Dose Intensity & Escalation**

| **Domain** | **Feature Name** | **Description** |
| --- | --- | --- |
| Dose Intensity | **max_rate** | Maximum NE dose achieved (µg/kg/min) |
| Escalation Kinetics | **t_to_max_d** | Days to reach maximum NE dose |
| Recovery Kinetics | **t50_after_peak_d** | Days from peak dose to 50% decline |
|  | **time_to_half_drop_d** | Alias of t50_after_peak_d |

**Linear Slopes (Signed)**

| **Domain** | **Feature Name** | **Description** |
| --- | --- | --- |
| Slopes | **slope_0_12h** | Linear slope from 0–12 h |
|  | **slope_12_48h** | Linear slope from 12–48 h |
|  | **slope_24_48h** | Linear slope from 24–48 h |
|  | **slope_48_72h** | Linear slope from 48–72 h |

**Slope Angles (Degrees)**

| **Domain** | **Feature Name** | **Description** |
| --- | --- | --- |
| Slope Angles | **slope_angle_0_12h** | Angle of slope 0–12 h |
|  | **slope_angle_12_48h** | Angle of slope 12–48 h |
|  | **slope_angle_24_48h** | Angle of slope 24–48 h |
|  | **slope_angle_48_72h** | Angle of slope 48–72 h |

**Angle Change Metrics**

| **Domain** | **Feature Name** | **Description** |
| --- | --- | --- |
| Turning Dynamics | **delta_angle_0_12_to_24_48** | Change in slope angle between early windows |
|  | **delta_angle_12_48_to_48_72** | Change in slope angle between mid windows |

**Threshold-Based Burden Metrics**

| **Domain** | **Feature Name** | **Description** |
| --- | --- | --- |
| Global Burden | **time_over_0.05** | Hours above 0.05 µg/kg/min |
|  | **time_over_0.10** | Hours above 0.10 µg/kg/min |
| High-Dose Burden | **time_over_0.45** | Hours above 0.45 µg/kg/min (avoids MIMIC-IV 0.50 truncation bias) |
| Windowed High-Dose Burden | **time_over_0.45_0_24h** | Time-over-0.45 within 0–24 h |
|  | **time_over_0.45_24_48h** | Time-over-0.45 within 24–48 h |
|  | **time_over_0.45_48_72h** | Time-over-0.45 within 48–72 h |
|  | **time_over_0.45_72_96h** | Time-over-0.45 within 72–96 h |

**Recovery & Withdrawal Indicators**

| **Domain** | **Feature Name** | **Description** |
| --- | --- | --- |
| Binary Recovery Features | **half_drop_by_day2** | ≥50% drop from peak by day 2 |
|  | **half_drop_by_day3** | ≥50% drop from peak by day 3 |
|  | **half_drop_by_day4** | ≥50% drop from peak by day 4 |
|  | **half_drop_by_day5** | ≥50% drop from peak by day 5 |

**Rebound Dynamics**

| **Domain** | **Feature Name** | **Description** |
| --- | --- | --- |
| Re-escalation | **rebound_cnt** | Count of post-peak rebound oscillations detected by combined strict + loose detectors |

**Clustering pipeline and K selection**

Trajectory phenotypes were derived using a two-stage clustering framework combining feature-based K-means with Dynamic Time Warping (DTW) refinement of the underlying time-series trajectories. In the first stage, K-means was applied to engineered norepinephrine (NA) exposure features after z-standardization. For each candidate K between 4 and 8, the algorithm performed 230 random initializations, and solutions were evaluated using between-cluster mortality separation, inertia, and seed-wise stability. Examination of inertia curves and seed-wise stability data (Supplement Table S3) showed clear stabilization beginning at K=5, with only marginal improvements beyond this range.

An important interpretive point is that the first stage of the framework defines the primary partition in the engineered exposure-feature space, and DTW serves as a refinement step rather than the sole driver of cluster formation. Accordingly, the resulting phenotypes should be interpreted as trajectory phenotypes derived within a prespecified, clinically motivated representation of norepinephrine exposure. In addition, the rule requiring at least 30 patients per final cluster was intended to reduce instability and overinterpretation of extreme-tail solutions, but it may also limit discovery of rare yet potentially meaningful micro-phenotypes. For transparency, the absorbed “Fulminant Shock” cluster is shown explicitly before merging.

**Figure S2. Elbow plot for K selection**


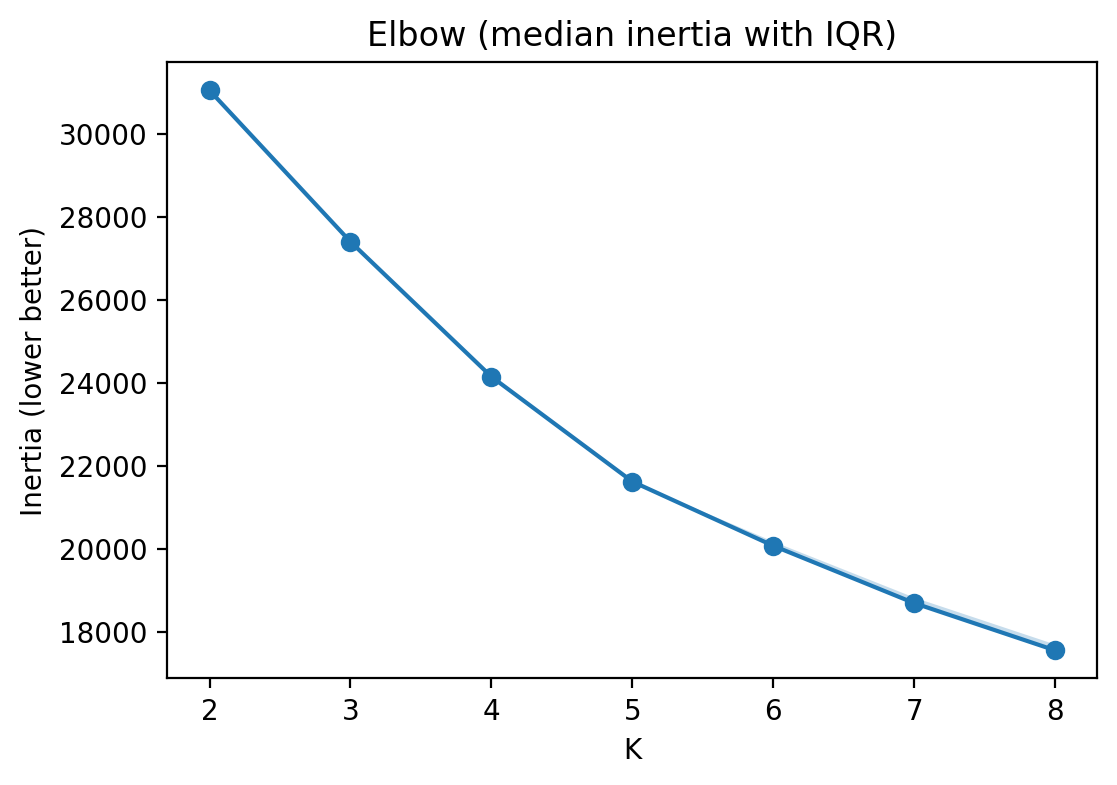


**Figure S2.** Elbow analysis demonstrating the relationship between the number of clusters (K) and the median within-cluster inertia across 230 random initializations of the K-means algorithm in the Sheba cohort*.* The plot shows a marked inflection between K=4 and K=5, with a smaller additional improvement at K=6 and progressively diminishing returns beyond this range. Both K=5 and K=6 provide substantially lower inertia than K=4, indicating improved within-cluster homogeneity. In the final model, K=5 was selected because it offered the optimal balance between stability, parsimony, and cluster interpretability; in addition, at K=6 one cluster failed to meet the pre-specified minimum-size criterion after DTW refinement and was therefore absorbed. This analysis supports the robustness of the five-phenotype solution used in the derivation cohort.

**Table S3**. **Inertia profiles across 230 seeds for candidate K values.**

| K | n_seeds | mean_inertia | sd_inertia | min_inertia | q1_inertia | median_inertia | q3_inertia | max_inertia |
| --- | --- | --- | --- | --- | --- | --- | --- | --- |
| 2 | 230 | 31165.38 | 868.6477 | 31049.5 | 31050.03 | 31050.03 | 31050.62 | 37851.31 |
| 3 | 230 | 27687.03 | 347.0461 | 27400.91 | 27436.65 | 27597.06 | 27843.42 | 29878.31 |
| 4 | 230 | 24423.26 | 539.9677 | 24150.58 | 24156.76 | 24163.29 | 24192.42 | 26716.12 |
| 5 | 230 | 22300.02 | 492.218 | 21618.95 | 21839.81 | 22213.29 | 22560.72 | 24710.24 |
| 6 | 230 | 20652.9 | 348.8285 | 20073.94 | 20348.8 | 20695.19 | 20825.2 | 21828.6 |
| 7 | 230 | 19313.73 | 397.6114 | 18682.11 | 19110.31 | 19244.88 | 19564.55 | 20795.81 |
| 8 | 230 | 18020.1 | 339.5082 | 17539.73 | 17727.07 | 17978.63 | 18210.64 | 18946.32 |

Across 230 K-means initializations per K, inertia decreased from 31,165 for K=2 to 18,020 for K=8, with the largest improvements observed between K=2–4 and progressively smaller gains thereafter (Δmean inertia: -3,478 from K=2-3, -3,264 from K=3-4, then -2,123, -1,647, -1,339, and -1,294 for higher K). For each K, the inertia distribution across seeds was narrow (small SD and Q1–Q3 range), indicating high stability with respect to random initialization and supporting a parsimonious choice of K≈5-6.

Although K=6 also produced a clinically coherent set of patterns, one of the six clusters (Cluster 1 in Figure S3) contained only 24 patients and therefore did not meet the prespecified minimum cluster-size threshold (≥30 patients). This cluster represented a “Fulminant Shock” micro-phenotype characterized by extremely high NA doses within the first 6 hours and 86% 90-day mortality. This cluster was absorbed into its nearest neighbor based on DTW trajectory similarity between cluster medoids. This process yielded five stable and clinically interpretable phenotypes in the Sheba derivation cohort. A possible explanation for the absence of this micro-phenotype in MIMIC-IV is reduced representation of very high early-dose norepinephrine exposures in that dataset. In direct communication, a member of the MIMIC team indicated that 0.5 µg/kg/min was the maximum norepinephrine dose allowed by the pharmacy/protocol at the source hospital. This may have limited the appearance of the extreme early-dose exposures that defined the fulminant-shock micro-phenotype in Sheba. In addition, differences in treatment-limitation practices across health systems may truncate the evolution of rapidly escalating vasopressor trajectories. These explanations should therefore be regarded as plausible contributors rather than direct conclusions from the present data.

**Figure S3. Noradrenaline trajectories for the development cohort, prior cluster absorption**


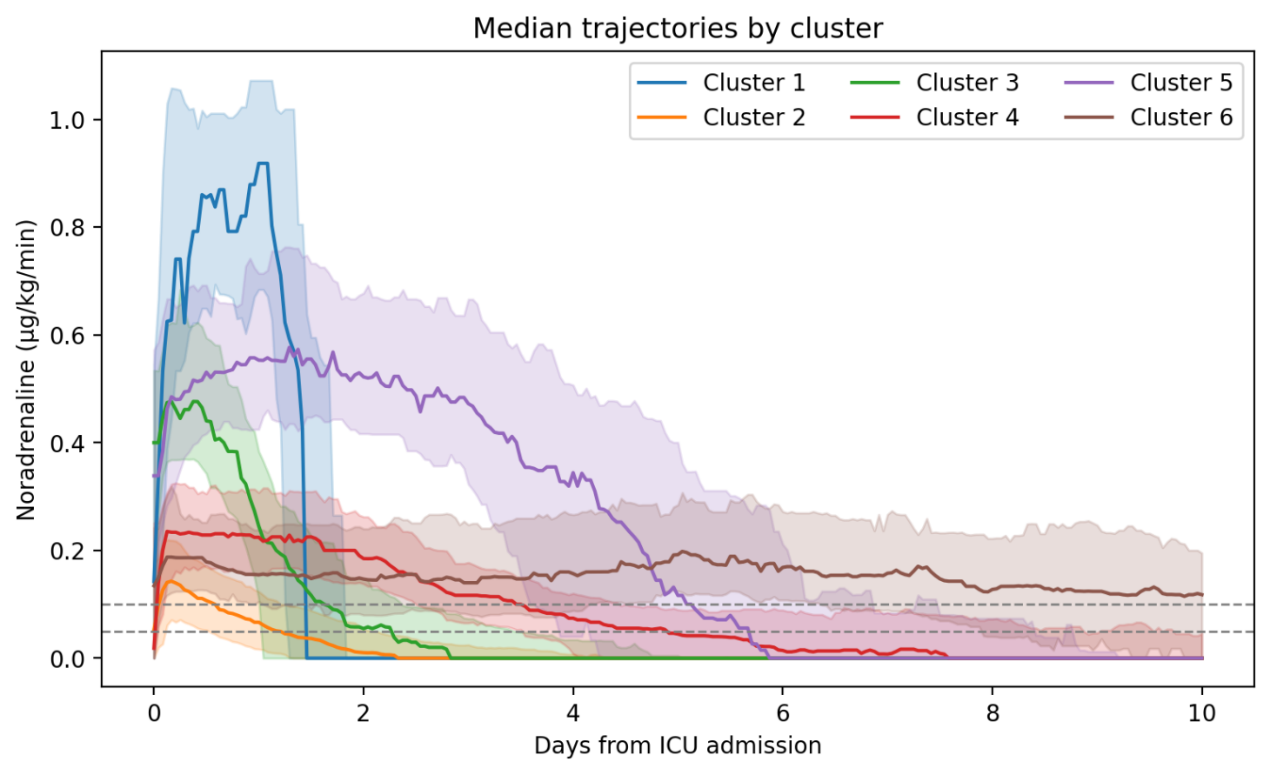
**Figure S3.** Median norepinephrine trajectories for the K=6 preliminary solution, Sheba cohort, prior to <30 patient cluster absorption. Shaded regions denote interquartile ranges. Cluster 1 is the absorbed small cluster.

**Phenotype structure in MIMIC-IV and cross-cohort harmonization**

When the same clustering pipeline (K-means on engineered NA features, DTW medoid refinement, absorption of undersized clusters, and renumbering by 96-hour landmark 90-day mortality) was applied to MIMIC-IV, it yielded six phenotypes. Compared with Sheba, the low-dose early-resolving pattern reproducibly split into two closely related subgroups: min-dose early resolving cluster (with extremely low and short lived NA dosage and low-dose early resolving cluster (which is quite similar to the Sheba low-dose early resolving cluster). Both clusters characterized by low NA exposure and rapid resolution.

For all cohort-specific descriptive and survival analyses, we retained each cohort’s native cluster structure (five phenotypes in Sheba, six in MIMIC-IV), with prognosis-based renumbering performed separately in each cohort.

However, for the early-phenotype prediction analysis, a common label space was required to allow the Sheba-trained model to be evaluated fairly in MIMIC-IV. To achieve this, the two low-dose, early-resolving MIMIC-IV phenotypes-which showed near-identical exposure profiles and both aligned with Sheba’s Low, Early Resolver class-were consolidated into a single unified “Minimal/Low, Early Resolver” phenotype, and the remaining MIMIC-IV phenotypes were mapped to their closest Sheba counterparts based on DTW trajectory similarity and feature profiles. This harmonization step was used only for the early-feature classification models and did not alter the underlying cohort-specific clustering or survival analyses.

After harmonization for early-phenotype prediction, the unified phenotype structure used for cross-cohort modeling consisted of the following five clinically interpretable groups:

*(1)* Minimal/Low, Early resolver*,
(2)* High, Early resolver*,
(3)* Intermediate, Gradual wean*,
(4)* High, Slow resolver*,
(5)* Intermediate, Non-resolver*.*

**Internal prognostic validation**

Internal discrimination of the landmark Cox models was assessed using a split-sample design. Test-set C-indices were computed at each prespecified landmark time between 24 and 144 hours. As shown in Supplement Figure S4, discrimination remained highly consistent across all windows (0.634–0.660), with the best performance observed at 48 hours and a similarly high value at 72 hours. Although a mild gradual decline was seen at later landmarks, no substantial degradation occurred, indicating that the direction and relative strength of covariate effects remained stable throughout the early course of shock. This temporal robustness supports the use of the 96-hour landmark as the primary analytic window in the main manuscript.

**Figure S4. Landmark Cox model discrimination across timepoints**


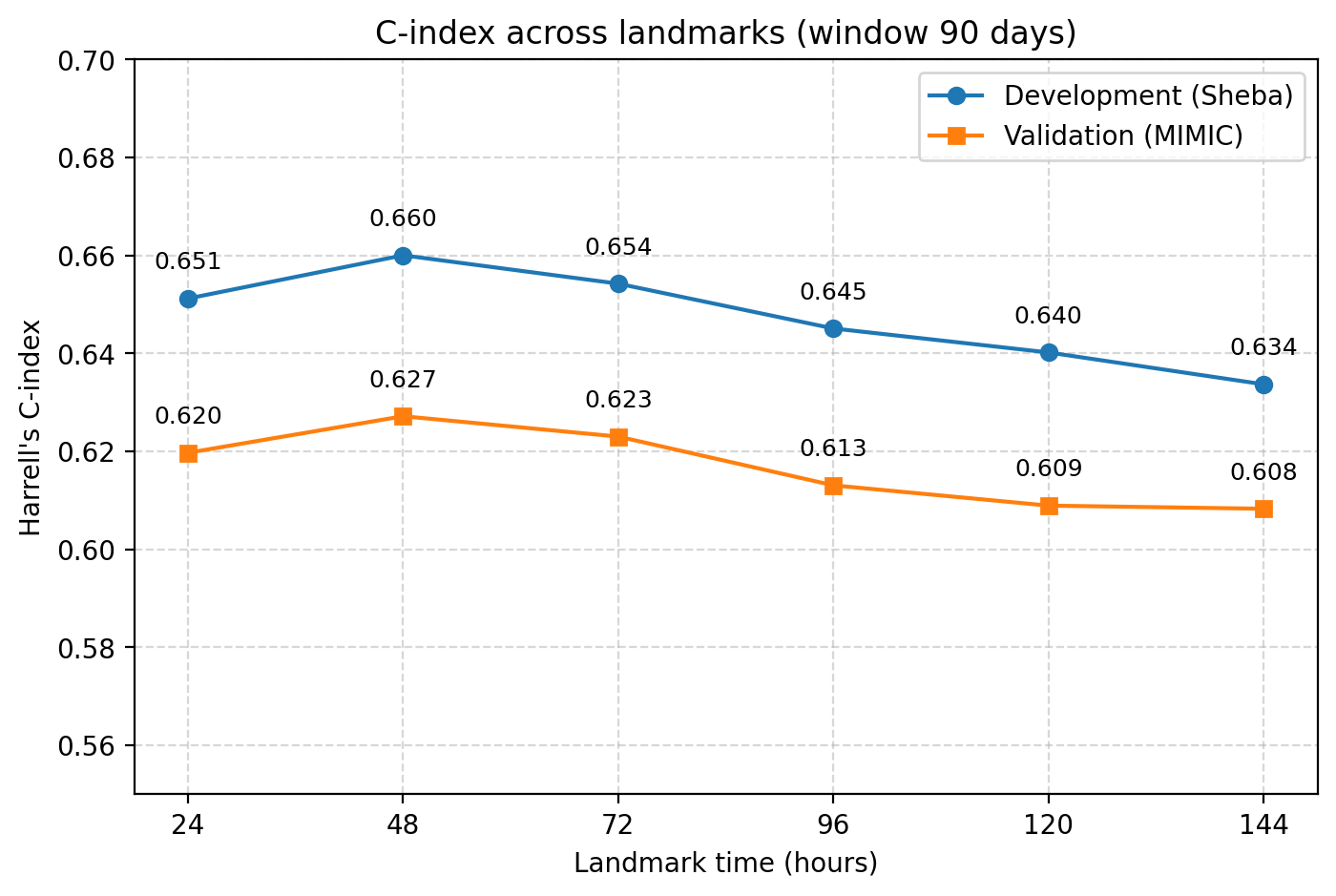


**Figure S4.** Harrell’s C-index for multivariable landmark Cox models evaluated at 24, 48, 72, 96, 120, and 144 hours after ICU admission in the derivation cohort (blue) and external validation cohort (orange). Each point represents the discrimination of a separate time-specific model using the same set of baseline covariates and norepinephrine trajectory features available at that landmark. C-index values in the derivation cohort ranged from 0.634 to 0.660 and remained highly stable across all windows, with the highest performance observed at 48 hours and only modest decline thereafter. The validation cohort showed similarly consistent performance (0.602–0.621).

**Sensitivity Analysis of Feature-Set Size for Early Phenotype Prediction**

**Rationale**

Because early-phenotype prediction relies on a reduced set of norepinephrine exposure-derived features, we conducted a structured sensitivity analysis to determine whether increasing the number of top Sheba-ranked features improved model performance, generalizability, or calibration. Five configurations using the top 8, 10, 12, 14, and 16 Sheba-ranked features were evaluated. All models were trained exclusively in Sheba and externally validated in MIMIC-IV using the same temperature-scaled multinomial framework described above.

Performance was assessed across the 24-, 48-, 72-, and 96-hour horizons using overall accuracy, macro-F1 score, Brier score, the fraction of patients receiving high-confidence predictions (P_max ≥ 0.80), and accuracy within the high-confidence subset.

**Supplement Table S4. Sensitivity analysis across different feature-set sizes (external validation: MIMIC-IV)**

Values represent means across the four prediction horizons (24–96 h).

| **Number of Features** | **Accuracy** | **Macro-F1** | **High-Confidence Fraction** | **Accuracy Among High-Confidence** | **Brier Score** |
| --- | --- | --- | --- | --- | --- |
| **8** | 0.8835 | 0.6366 | 0.891 | **0.931** | **0.1756** |
| **10** | 0.8830 | **0.6383** | 0.901 | 0.926 | 0.1806 |
| **12** | 0.8843 | **0.6383** | 0.906 | 0.924 | 0.1812 |
| **14** | 0.8845 | 0.6325 | 0.907 | 0.924 | 0.1816 |
| **16** | **0.8854** | 0.6356 | **0.909** | 0.924 | 0.1818 |

**Interpretation**

This sensitivity analysis revealed several consistent patterns:

**1. Overall accuracy changed minimally across configurations**

External accuracy varied only between 0.883–0.886, indicating that early-phenotype prediction is highly robust to the number of included features once the most informative exposure windows are present.

**2. Best discrimination (macro-F1) occurred with 10–12 features**

Models using 10 or 12 features achieved the highest macro-F1 scores (≈0.638), indicating slightly improved balance across minority phenotypes. Differences, however, were numerically small.

**3. Calibration and reliability were best with 8 features**

The 8-feature model demonstrated:

- The lowest Brier score (0.176), indicating superior calibration
- The highest accuracy among high-confidence predictions (~93.1%)
- Only a modest reduction in high-confidence coverage (≈89%) compared with larger models (~91%)

This combination-best calibration + best reliability when confident-suggests that adding additional features introduces mild overfitting to Sheba-specific patterns without improving external correctness.

**4. Increasing the feature count mainly increases confidence, not correctness**

High-confidence coverage rose from **89%⟶91%** when moving from 8→16 features, but accuracy within that subset decreased from **93.1%⟶92.4%**, reflecting a classic accuracy–confidence trade-off.

**5. Internal (Sheba) performance does not generalize linearly**

As expected, adding more features improved internal accuracy and macro-F1.
However, these gains did not translate to MIMIC-IV, so they reflect cohort-specific fit rather than generalizable signal.

**Conclusion of the Sensitivity Analysis**

Despite very small differences in overall accuracy, the 8-feature configuration provided the best external calibration, highest correctness among high-confidence predictions, and the most reliable behavior across horizons.

Therefore, the final early-phenotype prediction model used the top eight Sheba-ranked exposure features, with all larger feature sets reported as sensitivity analyses.

**Early-phenotype prediction model**

To evaluate whether norepinephrine (NE) trajectory phenotypes could be identified before the full 10-day exposure period had elapsed, we developed a series of horizon-limited supervised classification models restricted to information available within the first 24, 48, 72, and 96 hours after ICU admission. At each horizon, only exposure-derived variables fully computable within that window were included; these consisted of short-interval AUC components and global time-over-dose metrics. All continuous predictors were standardized using means and standard deviations estimated from the Sheba training partition.

Because the Sheba derivation cohort contained five final trajectory phenotypes whereas MIMIC-IV originally contained six, MIMIC-IV labels were harmonized to the unified five-phenotype structure prior to model training and validation. Classification was performed using class-balanced multinomial logistic regression with L2 regularization. To ensure stable and well-calibrated probability estimates across cohorts, we applied temperature scaling to the model logits, with the temperature parameter estimated on the Sheba development set and held fixed for all internal-test and external-validation predictions.

For each prediction horizon, a separate model was trained exclusively on the Sheba cohort, evaluated on an untouched Sheba test set, and subsequently applied without retraining to the harmonized MIMIC-IV cohort. Model performance was summarized using overall accuracy, macro-F1 score, Brier score, the proportion of patients receiving a high-confidence prediction (defined as maximum class probability P_max ≥ 0.80), and accuracy within this high-confidence subgroup.

**Supplement Table S5. Early phenotype-prediction performance using temperature-scaled probabilities (8-feature model)**

**Derivation Cohort (Sheba)**

| **Horizon (hours)** | **Accuracy** | **High-Confidence Coverage** | **Accuracy Among High-Confidence** |
| --- | --- | --- | --- |
| **24** | 0.852 | 0.749 | 0.940 |
| **48** | 0.892 | 0.776 | 0.948 |
| **72** | 0.901 | 0.798 | 0.961 |
| **96** | 0.852 | 0.753 | 0.964 |

**External Validation Cohort (MIMIC-IV)**

| **Horizon (hours)** | **Accuracy** | **High-Confidence Coverage** | **Accuracy Among High-Confidence** |
| --- | --- | --- | --- |
| **24** | 0.863 | 0.882 | 0.914 |
| **48** | 0.884 | 0.894 | 0.929 |
| **72** | 0.889 | 0.909 | 0.932 |
| **96** | 0.898 | 0.880 | 0.948 |

High-confidence predictions were defined as maximum class probability P_max ≥ 0.80.

**Interpretation**

Across both cohorts, the ability to anticipate each patient’s eventual norepinephrine-trajectory phenotype emerged early and remained stable as additional exposure data accumulated. Using only 24-hour features, overall accuracy reached 0.85 in the Sheba derivation cohort and 0.86 in the MIMIC-IV external cohort, with high-confidence subsets demonstrating 91-94% correctness.

By 48 hours, high-confidence coverage expanded to 78% of Sheba patients and 89% of MIMIC-IV patients, while accuracy within this subgroup remained high (93-95%). Performance improved modestly through 72–96 hours: accuracies rose to 0.90 in Sheba and 0.89-0.90 in MIMIC-IV, and high-confidence coverage reached 80% in Sheba and 88-91% in MIMIC-IV. Accuracy among high-confidence predictions remained consistently strong (approximately 93-96% across horizons).

Temperature scaling yielded well-calibrated probability estimates in both datasets, indicating that cross-cohort differences in vasopressor titration practices and documentation density did not materially compromise probability reliability.

Taken together, these findings demonstrate that NE trajectory phenotypes are highly learnable within the first 24–48 hours of septic shock resuscitation and that the model rapidly produces large, highly accurate, and well-calibrated high-confidence subsets in both derivation and external-validation cohorts. These results support early prognostic classification and dynamic stratification after the initial resuscitation window, but do not by themselves establish that acting on phenotype assignment improves outcomes.

**Supplement Table S6a. 24-hour prediction - MIMIC-IV (external validation)**

Overall accuracy ≈ **0.863** (from summary8)

| **True ↓ / Predicted →** | **Minimal/Low- Early** | **High-Early** | **Intermediate- Gradual wean** | **High-Slow** | **Intermediate- Non-resolver** |
| --- | --- | --- | --- | --- | --- |
| Minimal/Low | 7141 | 121 | 99 | 7 | 50 |
| High-Early | 57 | 382 | 180 | 24 | 3 |
| Intermediate GW | 510 | 1 | 366 | 1 | 94 |
| High-Slow | 3 | 15 | 105 | 43 | 14 |
| Non-resolver | 1 | 0 | 14 | 13 | 99 |

**Supplement Table S6b. 48-hour prediction – MIMIC-IV**

Overall accuracy ≈ **0.884**

| **True ↓ / Predicted →** | **Minimal/Low- Early** | **High-Early** | **Intermediate- Gradual wean** | **High-Slow** | **Intermediate- Non-resolver** |
| --- | --- | --- | --- | --- | --- |
| Minimal/Low | 7227 | 120 | 21 | 3 | 47 |
| High-Early | 54 | 406 | 166 | 19 | 1 |
| Intermediate GW | 392 | 6 | 476 | 1 | 97 |
| High-Slow | 0 | 6 | 111 | 57 | 6 |
| Non-resolver | 3 | 2 | 20 | 8 | 94 |

**Supplement Table S6c. 72-hour prediction – MIMIC-IV**

Overall accuracy ≈ **0.889**

| **True ↓ / Predicted →** | **Minimal/Low- Early** | **High-Early** | **Intermediate- Gradual wean** | **High-Slow** | **Intermediate- Non-resolver** |
| --- | --- | --- | --- | --- | --- |
| Minimal/Low | 7235 | 110 | 20 | 4 | 49 |
| High-Early | 63 | 420 | 152 | 7 | 4 |
| Intermediate GW | 355 | 5 | 495 | 0 | 117 |
| High-Slow | 0 | 3 | 116 | 56 | 5 |
| Non-resolver | 3 | 2 | 24 | 8 | 90 |

**Supplement Table S6d. 96-hour prediction – MIMIC-IV**

Overall accuracy ≈ **0.898**

| **True ↓ / Predicted →** | **Minimal/Low- Early** | **High-Early** | **Intermediate- Gradual wean** | **High-Slow** | **Intermediate- Non-resolver** |
| --- | --- | --- | --- | --- | --- |
| Minimal/Low | 7237 | 104 | 18 | 1 | 58 |
| High-Early | 62 | 421 | 155 | 3 | 5 |
| Intermediate GW | 321 | 4 | 525 | 0 | 122 |
| High-Slow | 0 | 5 | 119 | 49 | 7 |
| Non-resolver | 1 | 2 | 22 | 9 | 93 |

**Interpretation of confusion matrices**

Across all horizons, predictions for the Minimal/Low Early Resolver phenotype remained highly accurate. Misclassification was concentrated among the intermediate and high-slow phenotypes. The Intermediate Gradual Wean phenotype was frequently labelled as Minimal/Low and, to a lesser extent, Non-resolver; the reverse pattern was less common. The High-Slow phenotype was most often reassigned to Intermediate Gradual Wean. Importantly, the extreme phenotypes (Minimal/Low and Non-resolver) were rarely confused with one another. These matrices illustrate that the dominant phenotype families are well separated early, whereas intermediate trajectories exhibit greater early overlap.

The 48-hour alluvial visualization is presented in the main manuscript as Figure 5. Detailed horizon-specific confusion matrices are provided in Supplementary Tables S6a–d.

**Landmark feature construction and prevention of look-ahead bias.**

At each prespecified landmark (24, 48, 72, 96, 120, and 144 h after ICU admission), we constructed a risk set including only admissions alive and still under observation at that landmark time. Cox models were fit with time zero reset to the landmark, and follow-up continued to 90 days. Accordingly, analyses estimate prognosis conditional on survival to the landmark. To avoid look-ahead bias, all NA trajectory features were computed using only data available up to the landmark. For time-window AUC features, we applied an availability rule: a window was included in a given landmark model only if its entire interval had elapsed by the landmark (e.g., AUC 48–72 h was included only in landmarks ≥72 h; AUC 72–96 h only in landmarks ≥96 h). Continuous covariates were standardized (1 SD) within each landmark risk set to facilitate comparison of effect sizes across covariates.

Multivariable Cox analyses methods

To address the partial overlap between norepinephrine exposure and the cardiovascular component of the SOFA score, we performed additional sensitivity analyses at the 96-hour landmark using a modified SOFA score that excluded the cardiovascular component. In both the Sheba and MIMIC-IV cohorts, and for both 30-day and 90-day mortality, we fitted a simpler model adjusted for cluster, age, sex, APACHE II, and the modified non-cardiovascular SOFA score, as well as a more fully adjusted model that additionally accounted for use of other vasopressors, inotropic support, and renal replacement therapy by the landmark. These analyses were performed to confirm that the association between trajectory phenotype and mortality was not driven by inclusion of the cardiovascular SOFA component in the adjustment set.

Figure S5. **Association of 96-hour noradrenaline trajectory phenotypes with 30-day and 90-day mortality in the Sheba and MIMIC-IV cohorts**


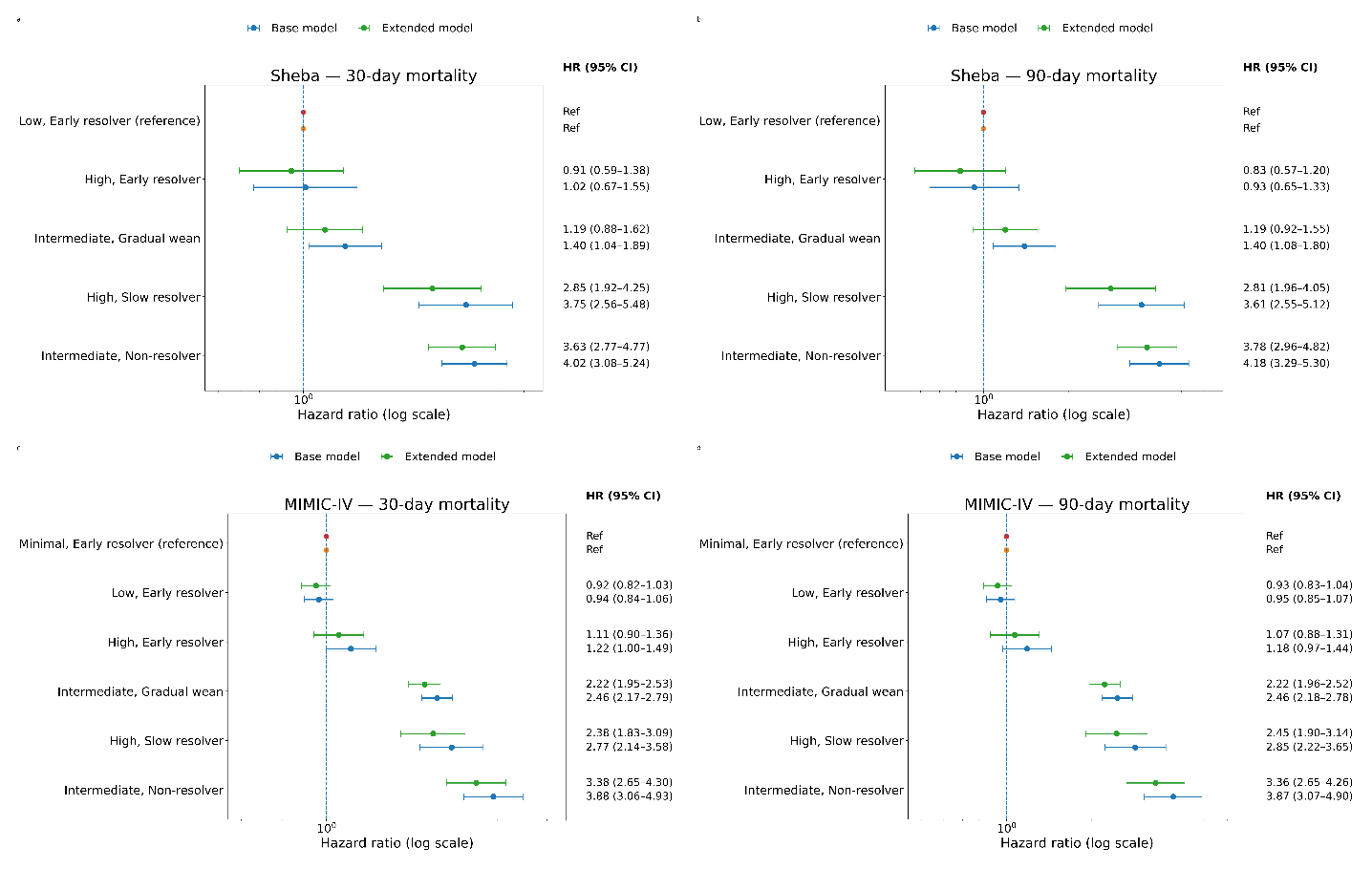


**Figure S5.** Forest plots show hazard ratios (HRs) and 95% confidence intervals for mortality according to named trajectory phenotypes, using the lowest-risk early-resolving phenotype in each cohort as the reference category. Panels a and b show the Sheba cohort for 30-day and 90-day mortality, respectively; panels c and d show the MIMIC-IV cohort for 30-day and 90-day mortality, respectively. Blue markers indicate the base model and green markers the extended model. The dashed vertical line marks HR=1. Estimates to the right of each panel are shown as HR (95% CI). Across both cohorts and both time horizons, progressively less favorable trajectories were associated with higher mortality, with the highest risks generally observed in the slow-resolving and non-resolving phenotypes.
